# Discordant Information on Blinding in Trial Registries and Published Research: A Systematic Review

**DOI:** 10.1001/jamanetworkopen.2024.52274

**Published:** 2024-12-26

**Authors:** Fengying Zhang, Yi Zhu, Shengmin Zhao, Qian Zhang, Huan Tao, Yunhong Wu, Pengli Jia

**Affiliations:** 1 Tibet Autonomous Region Clinical Research Center for High-Altitude Stress, Endocrinology and Metabolism Disease, Hospital of Chengdu Office of People’s Government of Tibetan Autonomous Region (Hospital.C.T.), Chengdu, China; 2 Proof of Concept Center, Eastern Hepatobiliary Surgery Hospital, Third Affiliated Hospital, Second Military Medical University, Naval Medical University, Shanghai, China; 3 Clinical Epidemiology and Evidence-based Medicine Research Center, West China Hospital, Sichuan University, Chengdu, Sichuan, China; 4 Department of Environmental and Occupational Health, Sichuan University West China School of Public Health, West China Fourth Hospital, Chengdu, Sichuan, China; 5 Department of Hematology, West China Hospital, Sichuan University, Chengdu, Sichuan, China; 6 School of Management, Shanxi Medical University, Taiyuan, China

## Abstract

**Importance:**

Blinding of individuals involved in randomized clinical trials (RCTs) can be used to protect against performance and biases, but discrepancies in the reporting of methodological features between registered protocols and subsequent trial publications may lead to inconsistencies, thereby reintroducing bias.

**Objective:**

To investigate inconsistency in blinding as reported in trial registries and publications.

**Data Sources:**

An exploratory dataset and a validation dataset were created. The exploratory dataset consisted of RCTs included in systematic reviews of adverse events from the SMART Safety database published between January 1, 2015, to January 1, 2020. The validation dataset was based on a literature search on PubMed for all registered RCTs published within the same time frame.

**Study Selection:**

Eligible RCTs for the exploratory dataset included were those that specified drug safety as the exclusive outcome and included at least 1 pairwise meta-analysis involving 5 or more RCTs of health care interventions. The validation dataset included a random selection of RCTs without restriction on outcome.

**Data Extraction and Synthesis:**

Preferred Reporting Items for Systematic Reviews and Meta-Analyses reporting guidelines were followed during data extraction. RCTs were matched to their registries and information on blinding was extracted from both the journal publication and trial registry. Extraction was performed by 1 author and cross-checked by 2 additional authors, with discrepancies resolved via consensus. The data analysis was conducted between July 2023 and January 2024.

**Main Outcomes and Measures:**

The primary outcome was inconsistency in blinding reports in the publication and the associated trial registry. Factors associated with the inconsistency were further investigated using multivariable logistic regression. The results were then compared with the validation dataset.

**Results:**

A total of 1340 RCTs were included, with a median (IQR) sample size of 338 (152-772) participants. Of these, 749 (55.90%) were multiregional, 1220 (91.04%) were multicenter, and 835 (62.31%) were prospectively registered. The most frequently studied condition was cancer, representing 472 trials (35.22%). In the exploratory dataset, 1080 trials (80.60%) had inconsistent reporting of blinding in their published trial registry. Higher odds of inconsistency were associated with trials conducted as single-center (OR, 2.84; 95% CI, 1.24-7.74; *P* = .02) or those focused on cancer (OR, 3.26; 95% CI, 2.04-5.38; *P* < .001). Evaluation of the 98 RCTs in the validation dataset revealed that 70 (71.43%) had inconsistencies between the published trial and its registries. The occurrence of inconsistencies was significantly higher in the exploratory dataset than the validation dataset (*P* = .03).

**Conclusions and Relevance:**

In this systematic review of RCTs, there were significant inconsistencies in the reporting of blinding between trial publications and their corresponding registries. These findings underscore the importance of maintaining consistency between registered protocols and published trial reports to ensure methodological transparency and minimize bias.

## Key Points

**Question:**

Does inconsistency exist in blinding status reports between publications and corresponding trial registries?

**Findings:**

In this systematic review of 1340 randomized clinical trials, 80.6% showed inconsistencies in blinding information between the publication and trial registry. Single-center trials and cancer-focused studies had notably higher odds of reporting inconsistencies.

**Meaning:**

These findings highlight the need for enhanced consistency between registered protocols and published reports to promote transparency and minimize potential bias in clinical trials.

## Teaser

This systematic review compared discrepancies between journal publications and trial registries on reporting of blinding in randomized clinical trials.

## Introduction

Randomized clinical trials (RCTs) are recognized as the preferred scientific approach for evaluating the treatment effects of health care interventions.^1^ In an RCT, participants are randomly allocated into intervention or control groups. The randomization procedure aims to create a level playing field for participants at the starting line, whereby participants of each group share almost equal features (ie, exchangeability), thus facilitating causal inference for any subsequent differences in outcomes between groups.^2^ The integrity of the randomization sequence is usually protected through the use of allocation concealment.^3^ Similarly, blinding of people involved in the trial can be used to protect against performance bias, detection bias, as well as the placebo or nocebo effect.^4^ These methodological features strengthen the interpretation of causality between interventions and outcomes by offering safeguards against biased treatment effect size estimates.

The undoubted value of comprehensive, transparent reporting of key methodological features is internationally acknowledged through reporting guidelines (eg, Consolidated Standards of Reporting Trials [CONSORT 2010]^5^ or Standard Protocol Items: Recommendations for Interventional Trials [SPIRIT 2013]^6^), inclusion in quality assessment tools,^7^ requirements for prospective trial protocol registration,^8^ and the development of trial registry platforms.^9^ Prospective trial registration aims to facilitate detection of any future bias that may creep in through protocol deviations as well as selective analysis and/or outcome reporting of subsequent trial findings.^10^ The US National Library of Medicine played an integral role in establishing the first international trial registry, ClinicalTrials.gov.^11^ In 2007, the US Food and Drug Administration began mandating that trials registered on the platform must make summarized trial results publicly available within 12 months of completion.^12^ Members of the public and health care professionals now have access to millions of trials, and are able to scrutinize essential information on study design and conduct.^13^

Discrepancies in the reporting of methodological features between registered protocols and subsequent trial publications may arise due to drafting errors or adjustments made during trial implementation.^14^ For instance, Hartung et al^15^ found that reporting discrepancies were common between the ClinicalTrials.gov results database and associated publications. Such discrepancies are problematic and risk jeopardizing evidence-based health care practice.^16^ We believe it is important to quantify the extent of consistency between the registered trial protocols and trial publications, particularly concerning critical methodological elements like blinding. Health care decision-makers need to be able to assess if the trial findings may have been affected by bias from lack of blinding. Equally, systematic reviewers often rely on blinding information when applying risk of bias (RoB) tools to evaluate study validity and synthesize evidence. Finally, there has been substantial debate in the recent literature surrounding the terminology and implementation of blinding, and it would be helpful to inform this debate with a quantitative empirical evaluation of how blinding is currently reported by 2 separate major sources of trial data. In this study, we evaluated the consistency of blinding status reporting in published trials and their corresponding trial registries using 2 datasets.

## Methods

### Study Design

This systematic review followed the Preferred Reporting Items for Systematic Reviews and Meta-Analyses (PRISMA) reporting guideline. We evaluated the reporting consistency of RCT blinding in 2 separate samples including (1) an exploratory dataset of RCTs included in systematic reviews of adverse events and (2) a validation dataset of a random sample of RCTs, without restriction on outcome, from the same time frame as the exploratory dataset.

### Data Sources and Search Strategy

The exploratory dataset utilized in this study was derived from the recently established SMART Safety empirical dataset.^17^ This dataset was established through a systematic literature search of PubMed of articles published from January 1, 2015, to January 1, 2020. Eligible systematic reviews were those that specified drug safety as the exclusive outcome and included at least 1 pairwise meta-analysis involving 5 or more RCTs of health care interventions.

Trial-level data of these eligible meta-analyses were extracted and verified by referencing the original trials using a double data extraction pattern. An adverse event was defined as “any untoward medical occurrence in a patient or subject in clinical practice,”^18^ which could be adverse effects, adverse reactions, injuries, or complications associated with any medical intervention.^19^ The representativeness of the search has been verified, and the detailed search strategy is outlined in eAppendix 1 in Supplement 1. Additionally, we restricted the selection of RCTs to exclude phase 1 trials because these trials do not necessarily use blinding; they typically involve a small number of participants to assess the safety of a drug or treatment, determine the optimal dosage, and establish the appropriate administration method.^20^

We developed a second independent dataset (ie, the validation dataset) to validate the results from the SMART Safety dataset. The validation dataset was based on a literature search on PubMed for all registered RCTs published within a specified time frame that matches the years of publication of RCTs included in the SMART Safety exploratory dataset. We targeted 100 trials for evaluation through stratified random sampling. The search strategy for the validation dataset is described in eAppendix 2 in Supplement 1.

### Definitions

The objective of this study was to identify if there was inconsistency in the reporting of blinding between published RCTs and their corresponding trial registries. We considered that full details on the implementation of blinding (ie, blinding present for 1 or more groups of people involved in the RCT, with specific details regarding exactly which of the following individuals were blinded) could involve as many as 6 distinct categories: (1) participants, (2) health care clinicians, (3) data collectors, (4) outcomes assessors, (5) data analysts, or (6) no blinding whatsoever (ie, open-label).

The aforementioned categories are based on the RoB 2 tool and the CONSORT 2010 and SPIRIT 2013 checklists,^5,6,21^ and we have presented detailed definitions of how we judged the reported blinding status (eTable 1 in Supplement 1). In a few cases, trial designers may have implemented blinding for individuals beyond the first 5 aforementioned categories; we did not make judgements on these other blinded individuals (eg, data monitoring committee members or manuscript writers) in our study assessments.^22,23^ Similarly, we could not determine blinding status when the roles of individuals in the trial were generically reported (eg, staff, all others, or no information) without further explanation or details.

### Data Extraction and Quality Control

To match the published RCTs to their registries, we checked the full texts for registration information. If the registration information was not available, we searched 4 main registries using the name of the principal author, intervention, control, sample size, and study design to identify matching entries on ClinicalTrials.gov, The International Clinical Trials Registry Platform, European Clinical Trials Register, and the International Standard Randomized Controlled Trial Number registry.^24,25,26,27^

The following data were extracted: (1) registration number, date of first registration, and start date of trial; (2) description of groups of individuals who were (or were not) blinded in the journal publication; (3) description of groups of individuals who were (or were not) blinded in the trial registries; and (4) other characteristics of the publications, including the year of publication, journal rank via Clarivate^28^ queried in February 2023, region, center, diseases classified by *International Classification of Diseases for Mortality and Morbidity Statistics, Eleventh Revision *(*ICD-11*),^29^ trial type, and funding. If any of these items were not provided in the article, efforts were made to obtain the missing information from the relevant registries. If the details regarding blinding were not available in the publications but were explicitly referenced to another source elsewhere, we proceeded to extract the details from the alternate source. In cases where neither the publication nor the registry provided the necessary information, it was considered missing data. In order to determine if a trial was prospectively registered, we looked at the first stated date of trial registration and the date the trial started; a trial registered before or within 1 month of the trial start date was regarded as prospective registration.^30^ To ensure comprehensive and systematic data collection, a structured data extraction form was developed and refined following pilot testing involving 10 clinical trials (eTable 2 in Supplement 1).

Data extraction was performed by 1 author (F.Y.) and cross-checked by 2 additional authors (P.L. and Z.Y.) to address any potential errors and ensure accuracy. Any discrepancies that arose were resolved through discussion among the authors to reach consensus, and in instances of persistent discrepancies, input from invited specialists was sought, with detailed records maintained for reference.

Given that the study periods of the exploratory and validation datasets overlapped, rigorous measures were taken to prevent the inclusion of the same RCT in both datasets. For each RCT, we compared key characteristics, including title, the trial registration number, intervention, and sample size, across the exploratory and validation datasets. This cross-checking process was designed to identify and remove any duplicate trials. If an RCT was found in both datasets, it was excluded from the validation dataset to ensure the independence of the 2 samples.

### Identification of Inconsistency

Three authors (F.Y., S.M., and Z.Q.) independently reviewed the information on blinding status reported in journal publications and compared it with the corresponding details provided in the trial registries. Reporting was judged as consistent when there was complete agreement between both the journal publication and trial registry regarding which individuals or groups in the trial were blinded, and which were not. Inconsistency was noted if 1 or more discrepancies were found in the blinding status across any category of individuals or groups involved in the trial. The specific criteria for these judgments are outlined in Table 1.

**Table 1.** The Result Scenarios for Determining Specific Criteria

Systmatic review categorization	Journal publication blinding status	Trial registry blinding status	Identification
Blinded^a^	Blinded	Fully reported	Consistent
	Blinded	Fully reported but ambiguous or unclear	
	Blinded	Partially reported	Inconsistent
	Blinded	Not blinded	
	Blinded	Not reported	
	Not reported	Not reported	
	Not reported	Blinded	
Open-label	Open-label	Open-label	Consistent
	Open-label (outcomes assessors)	Open-label (outcomes assessors)	
	Open-label	Open-label (outcomes assessors)	Inconsistent
	Open-label	Blinded	
	Open-label	Not reported	
	Open-label, outcomes assessors	Open-label	
	Not reported	Not reported	
	Not reported	Open-label	

^a^ Participants, health care clinicians, data collectors, outcomes assessors, or data analysts.

### Outcomes

The primary outcomes consisted of how often inconsistencies were found between journal publication and trial registry in the exact details of which individuals were blinded: (1) participants, (2) health care clinicians, (3) data collectors, (4) outcome assessors, (5) data analysts, and (6) none (ie, open-label). We used this information to construct a composite primary outcome: the overall proportion of trials affected by at least 1 (or more) inconsistencies of the 6 options between journal publications and corresponding trial registries in the reported blinding status of the aforementioned individuals. Finally, we measured the association of 7 prespecified trial characteristics with the likelihood of inconsistency in reporting blinding status.

### Statistical Analysis

Baseline characteristics of the study were summarized via counts and percentages for categorical variables, and medians and IQRs for continuous variables. The proportions of inconsistency were summarized separately according to which groups of individuals were reported to be blinded (or not). The χ^2^ test was used to compare the frequency of inconsistency between the exploratory dataset and the validation dataset.

To clarify the potential association of trial characteristics with the likelihood of the overall inconsistency, a multivariable logistic regression model was employed with the estimator as the odds ratio (OR). The following trial characteristics were considered: year of publication (2006-2010, 2011-2015, and 2016-2020), journal ranks by quartile (Q; Q1, Q2, Q3, Q4, or not included), study site (ie, Asia, America, Europe, or Africa), center (multicenter, single-center, or not reported), diseases (cancer; endocrine nutritional or metabolic; musculoskeletal; respiratory; digestive; mental, behavioral, or neurodevelopmental; and other diseases), funding (industry, academic, or not reported), and registration forms (prospective vs retrospective). Our rationale for stratification by these characteristics is that it allows for a more nuanced analysis by controlling for various factors, as well as the exploration of specific factors, thus leading to a deeper understanding of trends and potential sources of bias. The goodness-of-fit of the model was assessed using the Hosmer-Lemeshow test. Additionally, collinearity among the included variables was evaluated using the variance inflation factor.

Missing data occurred when insufficient information was reported in trial registries and/or published reports. For missing data, multiple imputation was employed using the chained equations method with random forest matching to generate 5 datasets with complete covariate information.

All statistical analyses were performed using R version 4.3.3 (R Project for Statistical Computing), with the packages mice version 3.16.0 for multiple imputation, and rms version 6.8-0 for regression. Statistical inferences were based on a 2-sided *t* test, with α = .05 as the significance level. Analyses were conducted from July 2023 to January 2024.

## Results

**Figure 1.**
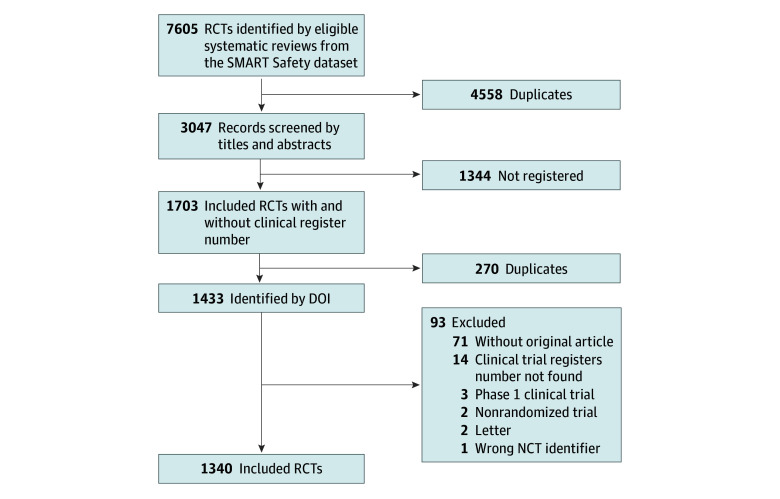
Diagram of Literature Screen DOI indicates digital object identifier; NCT, national clinical trial; RCT, randomized clinical trial.

A total of 2305 potentially relevant records were initially screened from the SMART Safety dataset for inclusion in the study. Among these, we were able to ascertain the registration information from journal publications of 1608 trials; a further 95 were matched to a registry entry following a search of the registry platforms. After excluding 270 duplicates and 93 studies that did not meet the inclusion criteria, a total of 1340 RCTs remained eligible for analysis in the exploratory dataset (Figure 1). A list of studies included in each dataset can be found in eTable 3 in Supplement 1.

Table 2 presents the baseline characteristics of the 1340 RCTs. The median (IQR) sample size of the RCTs was 338 (152-772) participants. Of these, 749 (55.90%) were multiregional trials, 1220 (91.04%) were multicenter trials, 835 (62.31%) were prospectively registered, and 1135 (84.70%) received funding from industry. In terms of diseases, the most common condition studied was cancer, which represented 472 of the 1340 RCTs (35.22%). Regarding publication characteristics, 896 of the 1340 trials (66.87%) were categorized as being published in Q1-ranked journals, and 765 (57.09%) were published between 2011 and 2015. Trial registries provided geographical details not mentioned in publications for 202 trials (15.07%) and included country-specific information for 154 trials (11.49%).

**Table 2.** Basic Characteristics of the Exploratory Dataset and the Validation Dataset

Basic characteristics	Studies, No. (%)	
	Exploratory dataset	Validation dataset
Trial type^a^		
Blinded	528 (39.40)	17 (17.35)
Open-label	209 (15.60)	18 (18.37)
Not clear	603(45.00)	63 (64.28)
Publication rank (Journal Citation Reports), quartile		
1	896 (66.87)	70 (71.43)
2	201 (15.00)	13 (13.27)
3	125 (9.33)	8 (8.16)
4	77 (5.75)	1 (1.02)
Not included	41 (3.06)	6 (6.12)
Region		
Europe only	158 (11.80)	35 (35.71)
Asia only	144 (10.75)	8 (8.16)
North America only	202 (15.07)	36 (36.74)
South America only and Africa only	5 (0.37)	2 (2.04)
Oceania only	11 (0.82)	2 (2.04)
Multiregion	749 (55.90)	12 (12.25)
Missing	71 (5.30)	3 (3.06)
Diseases (*ICD-11*)		
Cancer	472 (35.22)	18 (18.37)
Endocrine, nutritional, or metabolic diseases	274 (20.45)	9 (9.19)
Diseases of the musculoskeletal system or connective tissue	172 (12.84)	8 (8.16)
Diseases of the respiratory system	90 (6.72)	1 (1.02)
Diseases of the digestive system	74 (5.52)	2 (2.04)
Mental, behavioral, or neurodevelopmental disorders	55 (4.10)	8 (8.16)
Other diseases	203 (15.15)	52 (53.06)
Center		
Single	65 (4.85)	43 (43.88)
Multicenter	1220 (91.04)	52 (53.06)
Missing	55 (4.10)	3 (3.06)
Publication period		
2000-2005	25 (1.86)	NA
2006-2010	287(21.41)	13 (13.27)
2011-2015	765 (57.09)	38 (38.77)
2006-2020	263 (19.63)	47 (47.96)
Funding		
Industry	1135 (84.70)	61 (62.24)
Academic	205 (15.30)	37 (37.76)
Registration time		
Prospective	835 (62.31)	40 (40.82)
Retrospective	466 (34.78)	53 (54.08)
Indeterminable	8 (0.60)	NA
Missing	31 (2.31)	5 (5.10)

Abbreviations: *ICD-11, International Classification of Diseases for Mortality and Morbidity Statistics, Eleventh Revision*; NA, not applicable.

^a^ Blinded was defined as when both the trial and registry aligned in reporting that the treatment was concealed from either the participants, the health care clinicians, or both. Open-label was defined as when both the trial and registry explicitly stated that it was conducted as an open-label study. Any scenarios that did not fit within these definitions were categorized as not clear.

For the validation study, based on a literature search on PubMed, 47 615 records were obtained, and 100 records were randomly selected in terms of the year of publication, of which 2 were identified as non-RCTs. The details regarding the excluded studies can be found in eTable 4 in Supplement 1. The final validation dataset consisted of 98 RCTs; their baseline characteristics are presented in Table 2 and a list of the included studies can be found in eTable 3 in Supplement 1.

### Inconsistency in the Reporting of Blinding in the Exploratory Dataset

**Figure 2.**
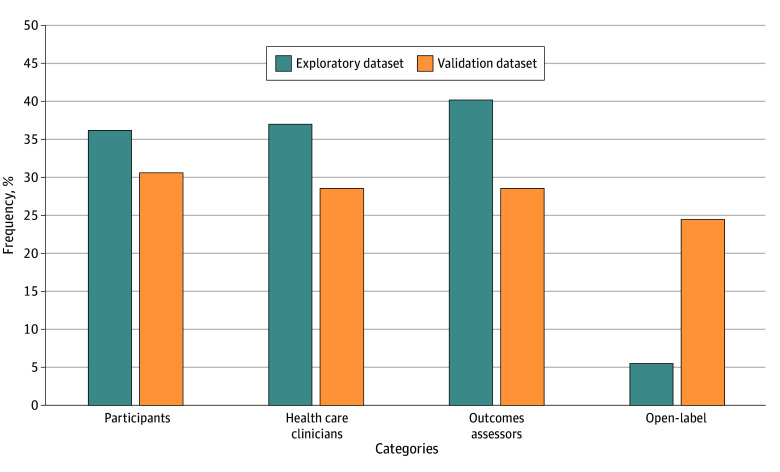
The Frequency of Discrepancies in Individual Blinding Status This figure displays the inconsistency between trial registries and publications regarding individual blinding statuses.

In the exploratory study of the 1340 included RCTs, we found that 1080 (80.60%) had 1 or more inconsistencies in reported blinding status between the registered protocol and subsequent trial publication. For individual blinding status, Figure 2 illustrates the inconsistency between trial registries and publications. Of the 1340 studies, 311 (23.30%) reported blinding in a broad, nonspecific manner, without specifying individuals’ blinding status. The inconsistency between registries and publications was moderate, ranging from 36.19% (485 studies) to 40.22% (539 studies), involving blinding of participants, health care clinicians, and outcomes assessors. The inconsistency for open-label RCTs was notably lower, at 5.52% (74 studies). Notably, the journal publications reported 8 cases of blinding of data collectors and 79 cases of blinding of data analysts; these were not reported on in the trial registries.

### Factors Associated With Inconsistent Reporting of Blinding Status

**Figure 3.**
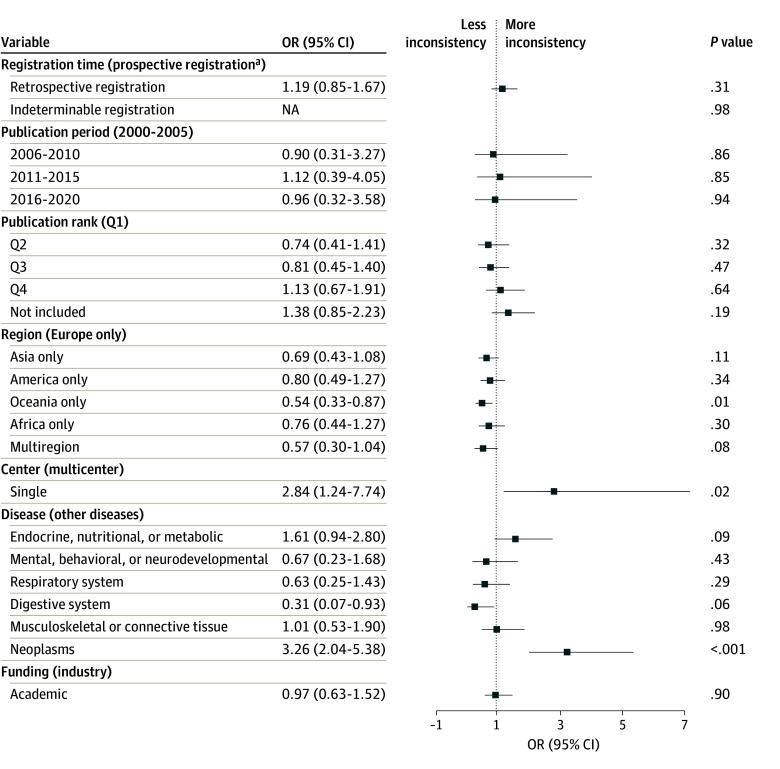
Associations of Trial Characteristics With Inconsistency in the Exploratory Dataset The figure presents the associations of trial characteristics with the likelihood of inconsistency between registries and publications in the exploratory dataset. Items in parentheses indicate the reference group. NA indicates not applicable; OR, odds ratio; Q, quartile. ^a^A trial registered before or within 1 month of the trial start date was regarded as prospective registration.

For the exploratory dataset, our multivariable regression analysis revealed that RCTs conducted as single-center had higher odds of inconsistency than those conducted as multicenter (OR, 2.84; 95% CI, 1.24-7.74; *P* = .02). Studies that focused on cancer had higher odds of inconsistency than those that focused on other diseases (OR, 3.26; 95% CI, 2.04-5.38; *P* < .001); of the 473 cancer-related publications, 327 (69.1%) did not specify blinding status. Meanwhile, RCTs conducted exclusively in Oceania regions had higher odds of inconsistency compared with studies conducted exclusively in Europe (OR, 0.54; 95% CI, 0.33-0.87; *P* = .01) (Figure 3).

### Inconsistency in the Reporting of Blinding in the Validation Dataset

In the validation study of the 98 RCTs, 70 (71.43%) exhibited 1 or more inconsistencies between the blinding status reported in trial registries vs publications. For individual blinding status, the validation dataset demonstrated fewer inconsistencies, with rates varying from 24.49% (24 studies) to 30.61% (30 studies) involving blinding of participants, health care clinicians, or outcomes assessors, or designation as open-label (Figure 2). Notably, the journal publications accounted for 1 case of blinding of data collectors and 3 cases of blinding of data analysts; these were not reported on in the trial registries. The occurrence of inconsistency in the exploratory dataset was 9.17 percentage points higher compared with the validation dataset (1080 of 1340 studies [80.60%] vs 70 of 98 studies [71.43%]; *P* = .03).

## Discussion

In this systematic review, we investigated inconsistencies in blinding reporting between published RCTs and their corresponding registries, finding a considerable degree of inconsistency. Specifically, RCTs identified from systematic reviews of harms exhibited a substantial level of inconsistency (80.60%), confirmed by our validation study of randomly selected RCTs (with no restriction to any specific topic or field), which similarly revealed a high degree of inconsistency (71.43%). The high prevalence of inconsistent reporting suggests a need for enhanced vigilance and improved transparency in the reporting of blinding information within clinical trial publications and their corresponding registries.

Several factors could explain the inconsistencies in blinding reporting. First, ambiguity may arise within the terminology used for describing the blinding status, especially when people involved in the RCT serve multiple roles (eg, data collection and outcome assessment). Moreover, broad or nonspecific terms, such as trial personnel or study team, may make it challenging to determine exactly who was blinded.^5,31^ ^32^ Second, some researchers may assume that using general blinding terminology suffices, without clarifying who specifically was blinded. Therefore, it is crucial to provide precise descriptions of those blinded, specifying whether and how each key role was blinded. In our study, 23.30% of publications (311 of 1340) reported blinding in a broad, nonspecific manner, without specifying individuals’ blinding status. Traditional blinding terminology alone may not offer unambiguous scientific communication; at the very least, it should be complemented by explicit reporting of exactly who was blinded. Third, methodological updates, including blinding status, may not be routinely reflected in public registries. Among our included studies, most of blinding data were obtained from ClinicalTrials.gov, where the fields for masking and masking description may be subject to varied interpretations by researchers and differ from journal editors’ requirements. Recommendations from authoritative publications suggested that broad terms, like investigators, should be avoided to eliminate ambiguity.^5,22^ These insights reveal potential contributors to the observed discordance between RCT publications and trial registries in blinding reports. To improve consistency and clarity, we emphasize the need of international consensus on definitions and terminology, along with enhanced reporting practices to ensure more accurate and standardized descriptions of blinding in both scientific publications and registries.

Our regression analysis highlights several factors associated with inconsistencies in blinding reporting. Single-center trials, often operating with smaller teams and fewer resources, may exhibit variability or less stringent documentation practices. These centers may also lack the administrative oversight necessary to ensure trial protocols are consistently updated and accurately reported across multiple platforms. In cancer research, ethical considerations frequently result in open-label trials (without placebo groups), which may contribute to the lack of blinding status reporting. Our analysis found that 69.1% of cancer-related publications (327 out of 473) did not specify blinding status. Additionally, the composition of our sample should be taken into account because we predominantly focused on RCTs reporting adverse events, which account for only approximately 43% of all published RCTs.^33^

Substantial efforts have been directed at streamlining the process of improving the transparency and comprehensiveness of evidence through the deployment of trial registers that host a substantial number of trials. Many of these trials can now share condensed data in a standardized manner with the public. This approach, gaining momentum for its potential to accelerate evidence synthesis, nonetheless reveals a level of inconsistency, as shown in our findings and corroborated by other research on trial characteristics and outcomes.^15,16,34,35,36^ On the positive side, our analysis shows that trial registries often include valuable information that publications lack. For example, registers provided geographical details for 15.07% of trials (202 of 1340 trials) not mentioned in papers, and 11.49% of trials (154 of 1340 trials) even included country-specific information, beyond simply listing the number of participating countries as is common in journal articles. While inconsistencies persist, registries continue to offer essential trial details that can enrich our understanding of study characteristics and outcomes.

Inconsistent blinding reports between trial registries and research articles bear important implications for RCTs outcomes, evidence-based practice, and decision-making. The first aspect involves transparency and study implementation. Transparency and study implementation are compromised when planned blinding procedures are not upheld or consistently reported. This discrepancy can skew the anticipated vs actual effects in trials, diminishing the credibility of trial outcomes. The second aspect is an examination of the assessment of RoB. Fluctuating blinding reports affect the assessment of RoB, potentially leading to incorrect RoB scores and, consequently, misleading conclusions in evidence quality assessments—especially problematic in quantitative synthesis models like the bias-adjusted model. The third is research integrity. The persistence of inconsistent reporting might lead researchers to deprioritize accurate reporting, promoting a concerning trend of lax reporting standards. Our study underscores the importance of addressing these inconsistencies to safeguard the integrity of clinical research, ensuring that evidence-based medical decisions rest on reliable, transparent data.

### Limitations

Our study possesses several limitations. First, we limited the analysis to systematic reviews from a 5-year publication window (2015-2020), which may exclude relevant evidence from earlier or more recent periods. Second, we imposed a restriction of at least 1 pairwise meta-analysis with 5 or more studies in our included systematic reviews. The limitations compromise the representativeness of the sample selection, potentially leading to false positive conclusions. Third, our analysis employed a logistic regression model to explore associations within a correlational cross-sectional design, limiting our ability to establish causal relationships. Fourth, due to insufficient methodological details available on the websites, we were unable to assess the association of additional factors with the RoB in the trial registers.

## Conclusions

In this systemic review of RCTs, we identified substantial challenges in the consistency of blinding as reported in published trials and their corresponding registries. Factors such as the disease focus, trial center, and geographic region were found to be associated with these discrepancies. Such inconsistencies may influence the assessment of RoB and could lead to misinformed health care practices. Urgent measures are needed to support authors in improving the consistency of blinding reports across both publications and trial registries in future randomized trials.

## Notes

Supplement 1. **eAppendix 1.** Search Strategy for Exploratory Dataset **eAppendix 2.** Search Strategy for Validation Dataset **eTable 1.** Descriptions of the Different Types of Blinding Status of Individuals **eTable 2.** Sample for Structured Data Extraction Form **eTable 3.** List of Included Studies in Exploratory Dataset and Validation Dataset **eTable 4.** List of Excluded Studies in Validation Dataset (With Reasons)

Supplement 2.
Data Sharing Statement
