# Supplementary material for: Discordant Information on Blinding in Trial Registries and Published Research: A Systematic Review
Source: JAMA Netw Open. 2024 Dec 26;7(12):e2452274. doi: 10.1001/jamanetworkopen.2024.52274 (PMC11672156; doi:10.1001/jamanetworkopen.2024.52274)
Supplement: Supplement 1. — eAppendix 1. Search Strategy for Exploratory Dataset eAppendix 2. Search Strategy for Validation Dataset eTable 1. Descriptions of the Different Types of Blinding Status of Individuals eTable 2. Sample for Structured Data Extraction Form eTable 3. List of Included Studies in Exploratory Dataset and Validation Dataset eTable 4. List of Excluded Studies in Validation Dataset (With Reasons) [file jamanetwopen-e2452274-s001.pdf]

Supplemental Online Content

Zhang F, Zhu Y, Zhao S, et al,. Discordant information on blinding in trial registries and published reseearch: a systematic review. *JAMA Netw. Open.* 2024;7(12):e2452274. doi:10.1001/jamanetworkopen.2024.52274

eAppendix 1. Search Strategy for Exploratory Dataset

eAppendix 2. Search Strategy for Validation Dataset

eTable 1. Descriptions of the Different Types of Blinding Status of Individuals

eTable 2. Sample for Structured Data Extraction Form

eTable 3. List of Included Studies in Exploratory Dataset and Validation Dataset

eTable 4. List of Excluded Studies in Validation Dataset (With Reasons)

This supplemental material has been provided by the authors to give readers additional information about their work.

© 2024 Zhang F et al. *JAMA Network Open*.

eAppendix 1: Search Strategy for exploratory dataset (PubMed, conducted on 28th-July, 2020)

1. "Systematic Reviews as Topic"[Mesh] OR "Systematic Review" [Publication Type] OR "Meta-Analysis as Topic"[Mesh] OR "Meta-Analysis" [Publication Type] OR "meta-analysis"[Title/Abstract] OR "meta analysis"[Title/Abstract] OR "systematic review"[Title/Abstract]
2. "randomized controlled trials as topic"[MeSH Major Topic] OR "clinical trials as topic"[MeSH Major Topic] OR "controlled clinical trials as topic"[MeSH Major Topic]
3. "randomized controlled trial*"[Title/Abstract] OR "controlled clinical trial*"[Title/Abstract] OR "clinical trial*"[Title/Abstract] OR "controlled trial*"[Title/Abstract] OR "trial*"[Title/Abstract]
4. "safety"[Title/Abstract] OR "harm*"[Title/Abstract] OR safe[Title/Abstract] OR poisoning[Title/Abstract] OR toxicity[Title/Abstract] OR tolerability[Title/Abstract] OR "complication*"[Title/Abstract] OR "adverse event*"[Title/Abstract] OR "adverse outcome*"[Title/Abstract] OR "untoward effect*"[Title/Abstract] OR "side effect*"[Title/Abstract] OR adverse n2 reaction[Title/Abstract]
5. #2 or #3
6. #1 AND #4 AND #5
7. Protocol[Title] OR overview [Title] OR "narrative review" [Title]
8. #6 NOT #7
9. (#8) AND (("2018/01/02"[Date - Publication]: "2020/01/01"[Date - Publication])) AND (humans[Filter]) Filters: Humans
10. (#8) AND (("2015/01/01"[Date - Publication]: "2018/01/01"[Date - Publication])) AND (humans[Filter]) Filters: Humans

© 2024 Zhang F et al. *JAMA Network Open*.

11. #9 or # 10

© 2024 Zhang F et al. *JAMA Network Open*.

eAppendix 2: Search Strategy for validation dataset (PubMed, conducted on 20th-December, 2023)

1. ("randomized controlled trial"[Publication Type] OR "controlled clinical trial"[Publication Type] OR "randomized"[Title/Abstract] OR "placebo"[Title/Abstract] OR "drug therapy"[MeSH Subheading] OR "randomly"[Title/Abstract] OR "trial"[Title/Abstract] OR "groups"[Title/Abstract]) NOT ("animals"[MeSH Terms] NOT "humans"[MeSH Terms])
2. 2000/01/01:2020/12/31[Print Publication Date]
3. "randomized controlled trial"[Publication Type]
4. "ISRCTN"[All Fields] OR "NCT"[All Fields] OR "ClinicalTrials.gov"[All Fields] OR "UMIN"[All Fields] OR "JapicCTI"[All Fields] OR "eudract"[All Fields] OR "ANZCTRN"[All Fields] OR "IRCT"[All Fields]
5. #1 and #2
6. #3 and #5
7. #4 and #6

© 2024 Zhang F et al. *JAMA Network Open*.

eTable 1. Descriptions of the different types of blinding status of individuals

Type of blinding status	Definition	Examples described in RCTs included
1. Participant	Participants are the individuals receiving the experimental interventions.	Participants, patients, subjects, individuals, the people receiving the treatment
2. Healthcare clinician	"*Healthcare clinicians include all personnel (for example, physicians, chiropractors, physiotherapists, nurses) who care for the participants during the trial.*" [23]	Healthcare providers, care providers, clinic staff, healthcare (medical management) practitioners, clinicians, caregivers, the people administering the treatment
3. Data Collector	"*Data collectors are the individuals who collect data on the trial outcomes.*"[23]	Data collector(s), persons collecting the data, data collection
4. Outcomes Assessor	Outcomes assessors are the individuals who evaluates the outcome(s) of interest.	Outcome adjudicators, clinical assessment outcomes assessor, assessment and central analysis, evaluators, the people assessing the outcomes
5. Data Analysts	Data analysts are the individuals conducting the analysis of data	Data analysts, data analysis, persons analysing (analysing) the data, persons who did the analysis, the people analysing the results/data
6. open-label	open-label means none of the participants nor Healthcare clinicians were blinded.	Open label, not blinded, unmasked.

© 2024 Zhang F et al. *JAMA Network Open*.

eTable 2. Sample for structured data extraction form

ID	Identifier of clinical trial registry	Citation information	Regis_form	Publication year	Journal Rank	Region	Center numbers	Disease	Funding	Trials	Website blinding application	Article blinding application
1	NCT00603512	Arthritis Care Res (Hoboken) 2011 Aug;63(8):1150-8	NI	2011	Q2	Japan	C19	Active rheumatoid arthritis (RA)	Pfizer	Not clear	Participant, Care Provider, Investigator, Outcomes Assessor	NI
2	NCT00527735	Ann Oncol, 2013, 24: 75-83	Pro	2013	Q1	7 countries	C32	Extensive-disease-small-cell lung cancer	Bristol-Myers Squibb	Not clear	Participant, Investigator	NI
3	NCT00528372	Diabet Med, 2015, 32: 531-41	Pro	2015	Q2	Canada, Mexico	C85	Type 2 diabetes	AstraZeneca, Bristol-Myers Squibb	Not clear	Participant, Investigator	Investigators, other clinic staff and participants
4	NCT00687297	J Thorac Oncol, 2013, 8: 1075-83	Retro	2013	Q1	United States	C26	Advanced non-small-cell lung cancer (NSCLC)	PrECOG, LLC.	Not clear	Participant, Care Provider, Investigator, Outcomes Assessor	Patients

© 2024 Zhang F et al. *JAMA Network Open*.

eTable 3. List of Included Studies in Exploratory Dataset and Validation Dataset

**Exclusion list**		
1	Exploratory Dataset	Arthritis Care Res (Hoboken) 2011 Aug;63(8):1150-8
2	Exploratory Dataset	Ann Oncol, 2013, 24: 75-83
3	Exploratory Dataset	Diabet Med, 2015, 32: 531-41
4	Exploratory Dataset	J Thorac Oncol, 2013, 8: 1075-83
5	Exploratory Dataset	Diabetes Obes Metab 2013 Oct;15(10):906-14
6	Exploratory Dataset	N Engl J Med 2011 Mar 3;364(9):818-28
7	Exploratory Dataset	N Engl J Med 2016; 375:209-219
8	Exploratory Dataset	N Engl J Med 2005 Dec 29;353(26):2747-57
9	Exploratory Dataset	Lancet Oncol 2010 Jan;11(1):21-8
10	Exploratory Dataset	Am J Psychiatry 2010 Jun; 167(6): 668–675
11	Exploratory Dataset	JAMA 2006 May 3;295(17):2003-17
12	Exploratory Dataset	JAMA 2013 Aug 7;310(5):488-95
13	Exploratory Dataset	N Engl J Med, 2006, 355: 1525-38
14	Exploratory Dataset	N Engl J Med, 2006, 355: 2542-50
15	Exploratory Dataset	N Engl J Med 2011 Oct 6;365(14):1273-83
16	Exploratory Dataset	J Clin Oncol 2009 Nov 20;27(33):5529-37
17	Exploratory Dataset	J Clin Oncol, 2012, 30: 2509-15
18	Exploratory Dataset	N Engl J Med, 2007, 357: 2666-76
19	Exploratory Dataset	N Engl J Med 2009 Jan 8;360(2):129-39
20	Exploratory Dataset	N Engl J Med 2005 Nov 3;353(18):1912-25

© 2024 Zhang F et al. *JAMA Network Open*.

21	Exploratory Dataset	N Engl J Med 2005 Dec 8;353(23):2462-76
22	Exploratory Dataset	N Engl J Med 2006 Feb 23;354(8):821-31
23	Exploratory Dataset	N Engl J Med 2005 Oct 20;353(16):1659-72
24	Exploratory Dataset	N Engl J Med 2008 May 22;358(21):2205-17
25	Exploratory Dataset	N Engl J Med 2007 Nov 1;357(18):1799-809
26	Exploratory Dataset	J Clin Oncol, 2013, 31: 3320-6
27	Exploratory Dataset	N Engl J Med 2007 May 3;356(18):1809-22
28	Exploratory Dataset	J Clin Oncol, 2011, 29: 2004-10
29	Exploratory Dataset	J Clin Oncol, 2011, 29: 1997-2003
30	Exploratory Dataset	J Clin Oncol 2009 May 20;27(15):2523-9
31	Exploratory Dataset	Lancet Oncol 2006 May;7(5):379-91
32	Exploratory Dataset	Gastroenterology 2008 Mar;134(3):688-95
33	Exploratory Dataset	N Engl J Med 2007 May 31;356(22):2271-81
34	Exploratory Dataset	Arthritis Rheum 2010 Jul;62(7):2101-8
35	Exploratory Dataset	J Clin Oncol, 2010, 28: 2137-43
36	Exploratory Dataset	N Engl J Med 2007; 356:125-134
37	Exploratory Dataset	J Clin Oncol 2009 Nov 20;27(33):5538-46
38	Exploratory Dataset	Lancet 2006 Oct 14;368(9544):1329-38
39	Exploratory Dataset	J Clin Oncol 2010 Aug 1;28(22):3605-10
40	Exploratory Dataset	Blood, 2010, 116: 5111-8
41	Exploratory Dataset	J Clin Oncol 2011 Jun 20;29(18):2459-65
42	Exploratory Dataset	Lancet 2008 Nov 22;372(9652):1809-18
43	Exploratory Dataset	Gut 2009 Jul;58(7):940-8 doi: 101136/gut2008159251
44	Exploratory Dataset	N Engl J Med 2006 Dec 28;355(26):2733-43

© 2024 Zhang F et al. *JAMA Network Open*.

45	Exploratory Dataset	N Engl J Med 2007; 357:2040-2048
46	Exploratory Dataset	JAMA 2012;307(13):1383-1393
47	Exploratory Dataset	Clin Colorectal Cancer 2014 Jun;13(2):100-9
48	Exploratory Dataset	Ann Intern Med 2005 Oct 18;143(8):559-69
49	Exploratory Dataset	Diabetologia 2007 Feb;50(2):259-67
50	Exploratory Dataset	Diabetes Care, 2011, 34: 2015-22
51	Exploratory Dataset	J Clin Oncol 2013 Jan 10;31(2):195-202
52	Exploratory Dataset	Am J Psychiatry 2010 Feb;167(2):181-9
53	Exploratory Dataset	J Clin Oncol, 2010, 28: 3617-22
54	Exploratory Dataset	J Clin Oncol 2013 Apr 10;31(11):1405-14
55	Exploratory Dataset	J Clin Oncol 2010 Apr 1;28(10):1756-65
56	Exploratory Dataset	Hum Vaccin 2011 Jul;7(7):768-75
57	Exploratory Dataset	Eur J Cancer, 2011, 47: 2331-40
58	Exploratory Dataset	Circulation 2008 Jun 10;117(23):3010-9
59	Exploratory Dataset	Lancet 2008 Jun 21;371(9630):2093-100
60	Exploratory Dataset	N Engl J Med 2007 May 10;356(19):1928-43
61	Exploratory Dataset	N Engl J Med 2007 May 10;356(19):1915-27
62	Exploratory Dataset	Rheumatology (Oxford), 2007, 46: 496-507
63	Exploratory Dataset	N Engl J Med 2006 Nov 23;355(21):2203-16
64	Exploratory Dataset	N Engl J Med 2010 Aug 19;363(8):711-23
65	Exploratory Dataset	Int J Clin Pract 2010 Apr;64(5):562-76
66	Exploratory Dataset	Diabetes Obes Metab 2007 Mar;9(2):194-205
67	Exploratory Dataset	Ann Rheum Dis 2008 Aug;67(8):1096-103
68	Exploratory Dataset	Lancet Oncol 2013 Dec;14(13):1326-36

© 2024 Zhang F et al. *JAMA Network Open*.

69	Exploratory Dataset	N Engl J Med, 2008, 359: 2456-67
70	Exploratory Dataset	J Clin Oncol 2013 Jan 20;31(3):359-64
71	Exploratory Dataset	N Engl J Med, 2010, 362: 1477-90
72	Exploratory Dataset	Curr Med Res Opin 2009 Jan;25(1):65-75
73	Exploratory Dataset	N Engl J Med 2007 Jan 11;356(2):115-24
74	Exploratory Dataset	Diabetes Obes Metab 2007 Mar;9(2):166-74
75	Exploratory Dataset	Diabet Med 2007 Sep;24(9):955-61
76	Exploratory Dataset	Diabetes Obes Metab 2008 Aug;10(8):675-82
77	Exploratory Dataset	Lancet 2008 Feb 2;371(9610):395-403
78	Exploratory Dataset	Horm Metab Res 2009 Dec;41(12):905-9
79	Exploratory Dataset	Curr Med Res Opin 2010 Oct;26(10):2339-46
80	Exploratory Dataset	N Engl J Med 2007 Nov 29;357(22):2237-47
81	Exploratory Dataset	Curr Med Res Opin 2008 Jun;24(6):1669-82
82	Exploratory Dataset	Lancet Oncol 2012 Mar;13(3):292-9
83	Exploratory Dataset	Diabetes Obes Metab 2010 May;12(5):442-51
84	Exploratory Dataset	Diabetes Care 2007 Aug;30(8):1979-87
85	Exploratory Dataset	Ann Intern Med 2007 Jun 19;146(12):829-38
86	Exploratory Dataset	N Engl J Med 2008 Jul 24;359(4):378-90
87	Exploratory Dataset	Diabetes Obes Metab 2009 Feb;11(2):157-66
88	Exploratory Dataset	Ann Rheum Dis 2008 Nov;67(11):1516-23
89	Exploratory Dataset	Clin Exp Rheumatol Jul-Aug 2016;34(4):625-33
90	Exploratory Dataset	Lancet, 2008, 371: 987-97
91	Exploratory Dataset	Arthritis Rheum 2008 Oct;58(10):2968-80
92	Exploratory Dataset	J Clin Oncol 2009 Jun 1;27(16):2630-7

© 2024 Zhang F et al. *JAMA Network Open*.

93	Exploratory Dataset	JAMA, 2010, 304: 2154-60
94	Exploratory Dataset	Ann Rheum Dis 2010; 69(1):88-96
95	Exploratory Dataset	J Clin Oncol 2013 Jan 20;31(3):373-9
96	Exploratory Dataset	J Clin Oncol 2008 May 1;26(13):2178-85
97	Exploratory Dataset	J Clin Oncol 2009 Jun 10;27(17):2823-30
98	Exploratory Dataset	Lancet, 2006, 367: 29-35
99	Exploratory Dataset	N Engl J Med 2006 Oct 19;355(16):1672-81
100	Exploratory Dataset	J Clin Oncol 2010 Feb 20;28(6):911-7
101	Exploratory Dataset	Lancet Oncol, 2012, 13: 1225-33
102	Exploratory Dataset	Circulation 2010 Mar 16;121(10):1176-87
103	Exploratory Dataset	J Clin Oncol 2009 Aug 10;27(23):3822-9
104	Exploratory Dataset	J Clin Oncol 2011 Aug 10;29(23):3120-5
105	Exploratory Dataset	BMJ, 2006, 332: 22-7
106	Exploratory Dataset	Diabet Med, 2013, 30: 1472-6
107	Exploratory Dataset	Diabetes Care 2009 Sep;32(9):1649-55
108	Exploratory Dataset	Endocr J 2010;57(5):383-94
109	Exploratory Dataset	Lancet, 2011, 377: 1846-54
110	Exploratory Dataset	Br J Cancer 2008 Sep 16;99(6):852-7
111	Exploratory Dataset	Obstet Gynecol 2009 Nov;114(5):999-1007
112	Exploratory Dataset	Gastroenterology 2014 Mar;146(3):681-688e1
113	Exploratory Dataset	JAMA 2006 Apr 5;295(13):1531-8
114	Exploratory Dataset	Diabetes Obes Metab 2009 Jun;11(6):571-8
115	Exploratory Dataset	Clin Ther 2007 Jun;29(6):1040-56
116	Exploratory Dataset	Ann Oncol 2013 Apr;24(4):1032-7

© 2024 Zhang F et al. *JAMA Network Open*.

117	Exploratory Dataset	Clin Ther 2007 Jun;29(6):1027-39
118	Exploratory Dataset	JAMA 2006 Jul 5;296(1):47-55
119	Exploratory Dataset	Lancet Oncol 2009 Nov;10(11):1063-9
120	Exploratory Dataset	JAMA 2006 Jul 5;296(1):64-71
121	Exploratory Dataset	Curr Med Res Opin 2007 Apr;23(4):793-801
122	Exploratory Dataset	JAMA 2006 Jul 5;296(1):56-63
123	Exploratory Dataset	Mod Rheumatol 2009;19(1):12-9
124	Exploratory Dataset	Scand J Rheumatol, 2008, 37: 142-50
125	Exploratory Dataset	J Clin Oncol 2012 May 20;30(15):1755-62
126	Exploratory Dataset	N Engl J Med 2008 Jun 12;358(24):2560-72
127	Exploratory Dataset	N Engl J Med, 2006, 355: 549-59
128	Exploratory Dataset	Am J Respir Crit Care Med 2008 Dec 1;178(11):1139-47
129	Exploratory Dataset	Lancet 2009 May 2;373(9674):1525-31
130	Exploratory Dataset	Lancet Oncol 2012 Jan;13(1):33-42
131	Exploratory Dataset	Curr Med Res Opin 2008 Jul;24(7):1931-41
132	Exploratory Dataset	N Engl J Med 2007 Nov 1;357(18):1810-20
133	Exploratory Dataset	Arthritis Rheum 2008 Nov;58(11):3319-29
134	Exploratory Dataset	Aliment Pharmacol Ther 2011 Jan;33(2):185-93
135	Exploratory Dataset	N Engl J Med 2007 Jul 19;357(3):239-50
136	Exploratory Dataset	N Engl J Med 2007 Jul 19;357(3):228-38
137	Exploratory Dataset	J Arthroplasty 2009 Jan;24(1):1-9
138	Exploratory Dataset	N Engl J Med, 2008, 359: 1225-37
139	Exploratory Dataset	Lancet, 2008, 372: 1174-83
140	Exploratory Dataset	N Engl J Med, 2008, 358: 1547-59

© 2024 Zhang F et al. *JAMA Network Open*.

141	Exploratory Dataset	N Engl J Med 2009; 360:1408-1417
142	Exploratory Dataset	J Clin Oncol 2015 Mar 1;33(7):692-700
143	Exploratory Dataset	J Clin Oncol 2011 May 20;29(15):2011-9
144	Exploratory Dataset	Lancet Neurol 2006 Jun;5(6):493-500
145	Exploratory Dataset	Clin Ther, 2008, 30: 2298-313
146	Exploratory Dataset	Lancet 2007 Sep 15;370(9591):949-56
147	Exploratory Dataset	Drugs Aging 2011 Jan 1;28(1):27-40
148	Exploratory Dataset	Arthritis Res Ther 2010;12(1):R7
149	Exploratory Dataset	J Rheumatol 2009 Sep;36(9):1991-9
150	Exploratory Dataset	Lancet 2005 Oct 8;366(9493):1279-89
151	Exploratory Dataset	Neurology, 2010, 75: 519-25
152	Exploratory Dataset	Ann Rheum Dis 2009 Jun;68(6):797-804
153	Exploratory Dataset	Epilepsia, 2013, 54: 89-97
154	Exploratory Dataset	Lancet 2008 Aug 2;372(9636):375-82
155	Exploratory Dataset	J Clin Oncol, 2011, 29: 884-9
156	Exploratory Dataset	Drugs 2008;68(14):1975-2000
157	Exploratory Dataset	Drugs 2009;69(5):549-65
158	Exploratory Dataset	Gut 2009 Oct;58(10):1354-62
159	Exploratory Dataset	Arthritis Rheum 2008 Sep 15;59(9):1270-8
160	Exploratory Dataset	Arthritis Rheum 2008 Apr;58(4):964-75
161	Exploratory Dataset	N Engl J Med 2009 Feb 5;360(6):563-72
162	Exploratory Dataset	Osteoporos Int 2009 Aug;20(8):1429-37
163	Exploratory Dataset	Drug Alcohol Depend 2014 Mar 1; 136: 100–107
164	Exploratory Dataset	Hypertension 2007 May;49(5):1047-55

© 2024 Zhang F et al. *JAMA Network Open*.

165	Exploratory Dataset	Lancet 2008 Jun 21;371(9630):2101-8
166	Exploratory Dataset	JAMA 2006 Dec 6;296(21):2572-81
167	Exploratory Dataset	JAMA 2008 Apr 2;299(13):1561-73
168	Exploratory Dataset	N Engl J Med, 2006, 354: 1685-97
169	Exploratory Dataset	Arthritis Rheum 2008 Jul;58(7):1981-91
170	Exploratory Dataset	Diabetes Care 2008 Jan;31(1):30-5
171	Exploratory Dataset	J Am Acad Dermatol, 2008, 58: 106-15
172	Exploratory Dataset	PLoS Clin Trials 2006 Jun;1(2):e11
173	Exploratory Dataset	Breast 2013 Oct;22(5):650-6
174	Exploratory Dataset	Arthritis Rheum 2011 Jun;63(6):1543-51
175	Exploratory Dataset	N Engl J Med, 2011, 364: 928-38
176	Exploratory Dataset	Lancet, 2008, 372: 1385-93
177	Exploratory Dataset	J Clin Oncol 2008 Sep 10;26(26):4244-52
178	Exploratory Dataset	Lancet, 2008, 372: 1394-402
179	Exploratory Dataset	J Clin Oncol, 2013, 31: 616-22
180	Exploratory Dataset	J Clin Oncol, 2009, 27: 2253-60
181	Exploratory Dataset	Ann Rheum Dis 2013 Jun;72(6):851-7
182	Exploratory Dataset	Gut 2011 Nov;60(11):1520-6
183	Exploratory Dataset	J Clin Oncol, 2011, 29: 1252-60
184	Exploratory Dataset	N Engl J Med 2009 Sep 17;361(12):1139-51
185	Exploratory Dataset	N Engl J Med, 2011, 365: 2473-83
186	Exploratory Dataset	Diabetes Care 2009 Apr;32(4):650-7
187	Exploratory Dataset	J Am Acad Child Adolesc Psychiatry 2011 Sep;50(9):903-14
188	Exploratory Dataset	Arthritis Rheum 2008 Nov;58(11):3402-12

© 2024 Zhang F et al. *JAMA Network Open*.

189	Exploratory Dataset	Arthritis Rheum 2009 Apr;60(4):976-86
190	Exploratory Dataset	Arthritis Rheum 2012 Aug;64(8):2504-17
191	Exploratory Dataset	Gastroenterology 2008 Oct;135(4):1130-41
192	Exploratory Dataset	Ann Oncol 2013 Sep;24(9):2382-9
193	Exploratory Dataset	J Clin Oncol 2014 Sep 20;32(27):2940-50
194	Exploratory Dataset	J Am Board Fam Med 2012 Jul-Aug;25(4):442-59
195	Exploratory Dataset	Lancet, 2008, 371: 1665-74
196	Exploratory Dataset	N Engl J Med 2007 Feb 22;356(8):775-89
197	Exploratory Dataset	N Engl J Med, 2017, 377: 338-351
198	Exploratory Dataset	Am J Cardiol 2008 Dec 1;102(11):1495-501
199	Exploratory Dataset	N Engl J Med 2006 Dec 7;355(23):2427-43
200	Exploratory Dataset	Lancet 2011 Jan 22;377(9762):321-31
201	Exploratory Dataset	Postgrad Med 2009 Mar;121(2):5-14
202	Exploratory Dataset	J Clin Oncol, 2011, 29: 4286-93
203	Exploratory Dataset	Circulation 2010 Jan 19;121(2):221-9
204	Exploratory Dataset	Lancet Oncol 2010 Sep;11(9):853-60
205	Exploratory Dataset	Chest 2011 Mar;139(3):591-599
206	Exploratory Dataset	Diabetes Obes Metab 2009 Dec;11(12):1145-52
207	Exploratory Dataset	Int J Clin Pract 2009 Jan;63(1):46-55
208	Exploratory Dataset	Diabetes Obes Metab 2009 Feb;11(2):167-76
209	Exploratory Dataset	J Clin Oncol 2007 Jul 1;25(19):2664-70
210	Exploratory Dataset	J Clin Oncol 2010 Apr 20;28(12):2024-31
211	Exploratory Dataset	Diabetes Res Clin Pract 2009 Jan;83(1):106-16
212	Exploratory Dataset	N Engl J Med 2009 Dec 10;361(24):2342-52

© 2024 Zhang F et al. *JAMA Network Open*.

213	Exploratory Dataset	Arthritis Rheum, 2011, 63: 3383-91
214	Exploratory Dataset	Diabetes Obes Metab 2011 Apr;13(4):348-56
215	Exploratory Dataset	Diab Vasc Dis Res 2011 Apr;8(2):125-35
216	Exploratory Dataset	Clin Infect Dis, 2008, 46: 1157-64
217	Exploratory Dataset	Lancet 2009 Jul 18;374(9685):210-21
218	Exploratory Dataset	Clin Drug Investig, 2008, 28: 625-34
219	Exploratory Dataset	Aliment Pharmacol Ther 2010 Oct;32(7):872-83
220	Exploratory Dataset	Oncology 2018;94(1):39-46
221	Exploratory Dataset	PLoS One 2013;8(3):e60094
222	Exploratory Dataset	Lancet, 2008, 371: 1675-84
223	Exploratory Dataset	J Neurol Neurosurg Psychiatry 2011 Sep;82(9):970-7
224	Exploratory Dataset	Br J Haematol 2010 May;149(3):426-35
225	Exploratory Dataset	Lancet Oncol 2010 Jul;11(7):619-26
226	Exploratory Dataset	J Am Coll Cardiol 2008 Dec 16;52(25):2127-34
227	Exploratory Dataset	Diab Vasc Dis Res 2011 Apr;8(2):150-9
228	Exploratory Dataset	Diabetol Metab Syndr 2012 Jul 24;4(1):36
229	Exploratory Dataset	J Pain Res 2013 Oct 25;6:743-53
230	Exploratory Dataset	Ann Rheum Dis 2007 Sep; 66(9): 1178–1183
231	Exploratory Dataset	Diabetes Care 2009 Jan;32(1):84-90
232	Exploratory Dataset	Pediatr Pulmonol, 2011, 46: 111-8
233	Exploratory Dataset	J Clin Oncol 2012 Jul 20;30(21):2585-92
234	Exploratory Dataset	J Gastrointest Cancer 2012 Dec;43(4):562-9
235	Exploratory Dataset	Clin Cancer Res 2012 Sep 1;18(17):4841-9
236	Exploratory Dataset	Eur J Cancer 2012 Nov;48(16):3082-92

© 2024 Zhang F et al. *JAMA Network Open*.

237	Exploratory Dataset	Lancet 2011 Mar 5;377(9768):813-22
238	Exploratory Dataset	Leuk Lymphoma 2013 Feb;54(2):321-8
239	Exploratory Dataset	Asia Pac J Clin Oncol 2012 Sep;8(3):232-43
240	Exploratory Dataset	N Engl J Med 2009 Sep 3;361(10):947-57
241	Exploratory Dataset	N Engl J Med 2010 Jun 24;362(25):2380-8
242	Exploratory Dataset	N Engl J Med 2011 Jun 30;364(26):2517-26
243	Exploratory Dataset	N Engl J Med 2011 Aug 25;365(8):689-98
244	Exploratory Dataset	Lancet 2016 May 14;387(10032):2008-16
245	Exploratory Dataset	Diabetes Obes Metab 2011 Jun;13(6):567-76
246	Exploratory Dataset	Lancet Oncol, 2012, 13: 154-62
247	Exploratory Dataset	J Clin Endocrinol Metab 2012 May;97(5):1615-22
248	Exploratory Dataset	N Engl J Med 2013 Feb 21;368(8):709-18
249	Exploratory Dataset	N Engl J Med 2008 Jun 26;358(26):2765-75
250	Exploratory Dataset	J Clin Oncol 2011 Mar 20;29(9):1125-32
251	Exploratory Dataset	Support Care Cancer 2014 Mar;22(3):679-87
252	Exploratory Dataset	Lancet 2008 Jul 5;372(9632):31-9
253	Exploratory Dataset	J Clin Oncol, 2010, 28: 3239-47
254	Exploratory Dataset	J Clin Oncol 2010 Feb 20;28(6):1061-8
255	Exploratory Dataset	Curr Med Res Opin 2008 Feb;24(2):537-50
256	Exploratory Dataset	Clin Cancer Res, 2012, 18: 555-67
257	Exploratory Dataset	Neuropsychopharmacology 2012 Mar;37(4):996-1004
258	Exploratory Dataset	Ann Rheum Dis 2011 May;70(5):747-54
259	Exploratory Dataset	J Clin Oncol 2010 Nov 1;28(31):4706-13
260	Exploratory Dataset	Lung Cancer 2010 Jun;68(3):420-6

© 2024 Zhang F et al. *JAMA Network Open*.

261	Exploratory Dataset	Breast Cancer Res Treat 2013 Feb;137(3):755-66
262	Exploratory Dataset	Lancet Oncol 2011 Apr;12(4):369-76
263	Exploratory Dataset	Diabetes Care 2009 Sep;32(9):1656-62
264	Exploratory Dataset	Epilepsia, 2016, 57: 210-21
265	Exploratory Dataset	Lancet, 2012, 379: 2270-8
266	Exploratory Dataset	Arthritis Rheum 2010 Apr;62(4):917-28
267	Exploratory Dataset	N Engl J Med 2008 Jun 26;358(26):2776-86
268	Exploratory Dataset	Lancet 2009 May 16;373(9676):1673-80
269	Exploratory Dataset	J Clin Oncol 2010 Nov 1;28(31):4697-705
270	Exploratory Dataset	N Engl J Med 2013 Sep 12;369(11):1023-34
271	Exploratory Dataset	J Clin Oncol 2011 Mar 10;29(8):1059-66
272	Exploratory Dataset	Ann Rheum Dis 2012 Apr;71(4):541-8
273	Exploratory Dataset	Ann Oncol, 2011, 22: 2057-2067
274	Exploratory Dataset	J Clin Psychopharmacol 2009 Aug;29(4):350-7
275	Exploratory Dataset	Lancet 2011 Jan 29;377(9763):393-402
276	Exploratory Dataset	Br J Cancer, 2011, 105: 618-27
277	Exploratory Dataset	N Engl J Med 2009 Aug 6;361(6):594-604
278	Exploratory Dataset	Respirology 2009 Apr;14(3):384-92
279	Exploratory Dataset	Invest New Drugs, 2012, 30: 1216-23
280	Exploratory Dataset	J Clin Oncol, 2015, 33: 4007-14
281	Exploratory Dataset	N Engl J Med 2011 Nov 24;365(21):1980-9
282	Exploratory Dataset	Lancet Oncol, 2013, 14: 88-96
283	Exploratory Dataset	Arch Intern Med 2009 Mar 23;169(6):616-25
284	Exploratory Dataset	N Engl J Med 2016 Dec 8;375(23):2246-2254

© 2024 Zhang F et al. *JAMA Network Open*.

285	Exploratory Dataset	N Engl J Med 2011 Jan 13;364(2):105-15
286	Exploratory Dataset	N Engl J Med 2007 Jul 5;357(1):28-38
287	Exploratory Dataset	Allergy Asthma Proc Jul-Aug 2010;31(4):280-9
288	Exploratory Dataset	Diabetes Obes Metab 2009 May;11(5):506-15
289	Exploratory Dataset	J Clin Oncol 2012 May 10;30(14):1620-7
290	Exploratory Dataset	Gut 2011 Jun;60(6):780-7
291	Exploratory Dataset	Eur J Cancer, 2013, 49: 1609-18
292	Exploratory Dataset	J Clin Oncol, 2013, 31: 1719-25
293	Exploratory Dataset	Am J Respir Crit Care Med 2011 Jul 1;184(1):92-9
294	Exploratory Dataset	J Child Adolesc Psychopharmacol 2011 Dec;21(6):581-8
295	Exploratory Dataset	Thorax 2010 Jun;65(6):473-9
296	Exploratory Dataset	Arthritis Rheum 2007 Dec;56(12):3928-39
297	Exploratory Dataset	Curr Med Res Opin 2010 May;26(5):1013-22
298	Exploratory Dataset	J Diabetes Investig 2011 Nov 30;2(6):441-7
299	Exploratory Dataset	J Clin Oncol 2012 Mar 20;30(9):921-9
300	Exploratory Dataset	Respiratory Medicine 2013;107(2):180-95
301	Exploratory Dataset	BMC Pulmonary Medicine 2012;12:67
302	Exploratory Dataset	Clin Ther 2013 Jul;35(7):950-66
303	Exploratory Dataset	Diabetes Obes Metab 2010 Feb;12(2):167-77
304	Exploratory Dataset	Diabetes Care 2010 Nov;33(11):2406-8
305	Exploratory Dataset	Diabetes Obes Metab 2012 Aug;14(8):745-52
306	Exploratory Dataset	Int J Clin Pract 2011 Feb;65(2):154-64
307	Exploratory Dataset	J Clin Oncol 2012 Oct 10;30(29):3596-603
308	Exploratory Dataset	J Clin Oncol 2010 Mar 20;28(9):1547-53

© 2024 Zhang F et al. *JAMA Network Open*.

309	Exploratory Dataset	J Clin Oncol, 2011, 29: 2215-22
310	Exploratory Dataset	N Engl J Med 2011 Sep 8;365(10):883-91
311	Exploratory Dataset	Int Clin Psychopharmacol 2016 Mar;31(2):61-8
312	Exploratory Dataset	J Clin Oncol 2012 Apr 1;30(10):1114-21
313	Exploratory Dataset	Gastroenterology 2012 Jul;143(1):62-69e4
314	Exploratory Dataset	N Engl J Med, 2012, 366: 310-20
315	Exploratory Dataset	Am J Cardiol 2009 Jun 15;103(12):1694-702
316	Exploratory Dataset	Cancer 2010 Sep 15;116(18):4256-65
317	Exploratory Dataset	Lancet 2008 Aug 9;372(9637):449-56
318	Exploratory Dataset	J Clin Oncol 2012 Jan 10;30(2):134-41
319	Exploratory Dataset	Lancet 2011 Dec 10;378(9808):2005-2012
320	Exploratory Dataset	Lancet, 2016, 387: 760-9
321	Exploratory Dataset	N Engl J Med 2011 Sep 15;365(11):981-92
322	Exploratory Dataset	Arthritis Rheum, 2012, 64: 970-81
323	Exploratory Dataset	JAMA 2013 Mar 27;309(12):1251-9
324	Exploratory Dataset	N Engl J Med 2010; 363:1889-1899
325	Exploratory Dataset	J Hematol Oncol 2012 Nov 29;5:71
326	Exploratory Dataset	J Clin Oncol 2011 Mar 10;29(8):1067-74
327	Exploratory Dataset	Clin Colorectal Cancer 2015 Jun;14(2):72-80
328	Exploratory Dataset	Clin Pharmacol Ther 2016 Oct;100(4):344-52
329	Exploratory Dataset	Respir Med 2012 Feb;106(2):257-68
330	Exploratory Dataset	Ann Allergy Asthma Immunol 2011 Sep;107(3):258-65e2
331	Exploratory Dataset	J Allergy Clin Immunol 2012 Aug;130(2):362-7e9
332	Exploratory Dataset	Ann Rheum Dis 2011 May;70(5):799-804

© 2024 Zhang F et al. *JAMA Network Open*.

333	Exploratory Dataset	Ann Rheum Dis 2010 Aug;69(8):1430-5
334	Exploratory Dataset	Ann Rheum Dis 2013 Jan;72(1):64-71
335	Exploratory Dataset	N Engl J Med 2010 Dec 23;363(26):2487-98
336	Exploratory Dataset	Eur J Cancer 2012 Mar;48(4):527-37
337	Exploratory Dataset	J Clin Lipidol Jul-Aug 2010;4(4):272-8
338	Exploratory Dataset	J Clin Oncol, 2015, 33: 4202-9
339	Exploratory Dataset	Phys Sportsmed 2010 Jun;38(2):19-28
340	Exploratory Dataset	N Engl J Med 2011 Feb 10;364(6):501-13
341	Exploratory Dataset	J Clin Oncol 2012 Jun 1;30(16):1989-95
342	Exploratory Dataset	J Clin Oncol 2010 Aug 1;28(22):3562-9
343	Exploratory Dataset	J Clin Oncol 2010 Dec 20;28(36):5240-6
344	Exploratory Dataset	Gastroenterology 2012 Aug;143(2):356-64e1
345	Exploratory Dataset	JAMA, 2010, 303: 1707-15
346	Exploratory Dataset	Ann Oncol, 2012, 23: 1693-9
347	Exploratory Dataset	Lancet Oncol 2014 Sep;15(10):1065-75
348	Exploratory Dataset	N Engl J Med 2008 May 22;358(21):2218-30
349	Exploratory Dataset	J Clin Oncol, 2012, 30: 34-41
350	Exploratory Dataset	J Clin Oncol, 2012, 30: 2039-45
351	Exploratory Dataset	Diabetes Care 2011 Mar;34(3):604-6
352	Exploratory Dataset	J Clin Oncol 2013 Aug 10;31(23):2870-8
353	Exploratory Dataset	Postgrad Med, 2011, 123: 220-7
354	Exploratory Dataset	N Engl J Med 2012 Nov 1;367(18):1714-23
355	Exploratory Dataset	N Engl J Med 2012 Apr 5;366(14):1287-97
356	Exploratory Dataset	N Engl J Med 2010 Dec 23;363(26):2499-510

© 2024 Zhang F et al. *JAMA Network Open*.

357	Exploratory Dataset	Clin Cancer Res 2015 Aug 15;21(16):3610-8
358	Exploratory Dataset	Clinical Research and Regulatory Affairs 2010;27:87–95
359	Exploratory Dataset	J Clin Psychiatry 2008 Jan;69(1):149-59
360	Exploratory Dataset	J Crohns Colitis 2012 Mar;6(2):160-73
361	Exploratory Dataset	Mod Rheumatol 2013 Jul;23(4):623-33
362	Exploratory Dataset	J Clin Oncol 2012 Sep 1;30(25):3084-92
363	Exploratory Dataset	Osteoarthritis Cartilage, 2012, 20: 350-356
364	Exploratory Dataset	Diabetes Obes Metab 2010 Mar;12(3):252-61
365	Exploratory Dataset	Ann Oncol 2015 Feb;26(2):325-32
366	Exploratory Dataset	Nutr J, 2008, 7: 9
367	Exploratory Dataset	Clinical Research and Regulatory Affairs 2011;28(1): 14–21
368	Exploratory Dataset	Lancet 2010 Mar 6;375(9717):807-15
369	Exploratory Dataset	Allergy Asthma Proc Mar-Apr 2011;32(2):127-36
370	Exploratory Dataset	Invest New Drugs 2013 Oct;31(5):1345-54
371	Exploratory Dataset	J Clin Oncol 2012 Apr 1;30(10):1122-8
372	Exploratory Dataset	Ann Oncol 2014 Feb;25(2):409-15
373	Exploratory Dataset	J Clin Oncol 2012 Jun 10;30(17):2070-8
374	Exploratory Dataset	J Clin Oncol 2013 Apr 1;31(10):1341-7
375	Exploratory Dataset	Lancet Oncol 2013 Jul;14(8):697-710
376	Exploratory Dataset	J Clin Oncol 2012 Aug 10;30(23):2829-36
377	Exploratory Dataset	J Thorac Oncol, 2014, 9: 1154-61
378	Exploratory Dataset	J Clin Oncol 2013 Jul 10;31(20):2586-92
379	Exploratory Dataset	Clin Ther 2009 Jan;31(1):177-89
380	Exploratory Dataset	Epilepsia, 2014, 55: 57-66

© 2024 Zhang F et al. *JAMA Network Open*.

381	Exploratory Dataset	J Cancer Res Clin Oncol 2013 Dec;139(12):2047-56
382	Exploratory Dataset	Lancet Oncol 2011 Mar;12(3):256-62
383	Exploratory Dataset	J Clin Oncol 2014 Mar 10;32(8):760-7
384	Exploratory Dataset	Clin Cancer Res 2010 Feb 15;16(4):1307-14
385	Exploratory Dataset	J Shoulder Elbow Surg, 2013, 22: 584-94
386	Exploratory Dataset	Int J Clin Pract 2009 Apr;63(4):547-59
387	Exploratory Dataset	Diabetes Obes Metab 2011 Jul;13(7):644-52
388	Exploratory Dataset	Lancet Oncol, 2013, 14: 1077-1085
389	Exploratory Dataset	Lancet Oncol 2013 Nov;14(12):1183-92
390	Exploratory Dataset	Haematologica, 2014, 99: 1343-9
391	Exploratory Dataset	Gastroenterology 2014 Jan;146(1):85-95; quiz e14-5
392	Exploratory Dataset	Gastroenterology 2014 Jan;146(1):96-109e1
393	Exploratory Dataset	Aliment Pharmacol Ther 2015 Sep;42(5):504-14
394	Exploratory Dataset	Epilepsia, 2014, 55: 47-56
395	Exploratory Dataset	J Clin Oncol 2016 Apr 1;34(10):1034-42
396	Exploratory Dataset	Mol Autism 2012 Dec 5;3(1):16
397	Exploratory Dataset	Arch Gen Psychiatry 2011 Dec;68(12):1195-206
398	Exploratory Dataset	Lancet Oncol 2009 Jan;10(1):25-34
399	Exploratory Dataset	Clin Cancer Res 2013 May 15;19(10):2745-54
400	Exploratory Dataset	Ann Oncol 2012 Apr;23(4):933-41
401	Exploratory Dataset	Eur J Cancer 2011 Sep;47(14):2117-27
402	Exploratory Dataset	Drug Saf 2009;32(9):787-800
403	Exploratory Dataset	Circ J 2013;77(3):632-8
404	Exploratory Dataset	Circ J 2012;76(9):2104-11

© 2024 Zhang F et al. *JAMA Network Open*.

405	Exploratory Dataset	Diabetes Obes Metab 2015 Jan;17(1):98-101
406	Exploratory Dataset	N Engl J Med 2011 Mar 3;364(9):806-17
407	Exploratory Dataset	J Clin Oncol 2013 May 1;31(13):1640-8
408	Exploratory Dataset	Gastroenterology 2016 Feb;150(2):380-8e4
409	Exploratory Dataset	Cancer Biother Radiopharm 2009 Apr;24(2):175-80
410	Exploratory Dataset	Child Adolesc Psychiatry Ment Health 2009 Jun 9;3(1):17
411	Exploratory Dataset	Lancet Oncol 2015 Feb;16(2):208-20
412	Exploratory Dataset	Epilepsia, 2014, 55: 38-46
413	Exploratory Dataset	JAMA Psychiatry 2013 May;70(5):522-33
414	Exploratory Dataset	J Hepatol 2012 May;56(5):1097-1103
415	Exploratory Dataset	Am J Kidney Dis 2013 Apr;61(4):579-87
416	Exploratory Dataset	Diabetes Care 2013 May;36(5):1067-73
417	Exploratory Dataset	N Engl J Med 2011 Feb 10;364(6):514-23
418	Exploratory Dataset	Diabetes Care 2013 May;36(5):1304-11
419	Exploratory Dataset	Palliat Med, 2012, 26: 50-60
420	Exploratory Dataset	Cancer Chemother Pharmacol 2012 May;69(5):1197-204
421	Exploratory Dataset	Gastroenterology 2014 Feb;146(2):442-52e1
422	Exploratory Dataset	Eur J Cancer 2013 Sep;49(13):2823-31
423	Exploratory Dataset	Lancet Oncol, 2013, 14: 760-8
424	Exploratory Dataset	J Asthma 2016 Oct;53(8):783-9
425	Exploratory Dataset	Congest Heart Fail May-Jun 2010;16(3):111-7
426	Exploratory Dataset	J Clin Oncol 2012 Jun 10;30(17):2046-54
427	Exploratory Dataset	Diabetes Care 2010 Oct;33(10):2217-24
428	Exploratory Dataset	Lancet Oncol, 2013, 14: 933-42

© 2024 Zhang F et al. *JAMA Network Open*.

429	Exploratory Dataset	BMC Med 2013 Feb 20;11:43
430	Exploratory Dataset	Ann Rheum Dis, 2012, 71: 198-205
431	Exploratory Dataset	J Clin Oncol, 2012, 30: 3640-7
432	Exploratory Dataset	Lancet Oncol 2015 Feb;16(2):187-99
433	Exploratory Dataset	Am J Cardiol 2010 Nov 1;106(9):1255-63
434	Exploratory Dataset	Ann Rheum Dis, 2015, 74: 694-702
435	Exploratory Dataset	Lancet Oncol 2008 Jan;9(1):39-44
436	Exploratory Dataset	Lancet Oncol 2012 Sep;13(9):897-905
437	Exploratory Dataset	J Clin Oncol 2013 Jul 1;31(19):2485-92
438	Exploratory Dataset	J Thromb Haemost 2012 May;10(5):799-806
439	Exploratory Dataset	Ann Oncol 2012 Nov;23(11):2799-2805
440	Exploratory Dataset	Rheumatology (Oxford) 2012 Jul;51(7):1226-34
441	Exploratory Dataset	Lancet Oncol 2012 Jan;13(1):25-32
442	Exploratory Dataset	Lancet Oncol 2015 Feb;16(2):221-32
443	Exploratory Dataset	J Clin Oncol, 2011, 29: 3968-76
444	Exploratory Dataset	Ann Rheum Dis 2009 Jun;68(6):805-11
445	Exploratory Dataset	Eur J Cancer 2013 Oct;49(15):3111-21
446	Exploratory Dataset	Arthritis Rheum 2012 Mar;64(3):617-29
447	Exploratory Dataset	Lancet Oncol 2014 Jul;15(8):819-28
448	Exploratory Dataset	Lancet 2012 Feb 18;379(9816):633-40
449	Exploratory Dataset	J Natl Cancer Inst 2014 Jul 16;106(7):dju151
450	Exploratory Dataset	Lancet Oncol, 2010, 11: 521-9
451	Exploratory Dataset	J Clin Oncol, 2012, 30: 3499-506
452	Exploratory Dataset	Ann Oncol 2017 Oct 1;28(10):2429-2435

© 2024 Zhang F et al. *JAMA Network Open*.

453	Exploratory Dataset	Inflamm Bowel Dis 2015 Jun;21(6):1329-40
454	Exploratory Dataset	Lancet 2013 Mar 16;381(9870):918-29
455	Exploratory Dataset	N Engl J Med 2015 Feb 19;372(8):724-34
456	Exploratory Dataset	N Engl J Med 2012 Jan 12;366(2):109-19
457	Exploratory Dataset	Am J Addict Sep-Oct 2010;19(5):401-8
458	Exploratory Dataset	N Engl J Med, 2012, 366: 299-309
459	Exploratory Dataset	Ann Surg Oncol, 2014, 21: 2517-24
460	Exploratory Dataset	Lancet Oncol 2012;13:135-44
461	Exploratory Dataset	Eur Respir J 2011 Feb;37(2):273-9
462	Exploratory Dataset	J Crohns Colitis 2013 Apr;7(3):239-47
463	Exploratory Dataset	Eur J Cancer, 2013, 49: 2633-42
464	Exploratory Dataset	Int J Clin Pract 2013 Apr;67(4):307-16
465	Exploratory Dataset	J Diabetes Investig, 2011, 2: 210-7
466	Exploratory Dataset	Clin Ther 2011 Apr;33(4):465-77
467	Exploratory Dataset	Arthritis Rheum 2011 Jul;63(7):1782-92
468	Exploratory Dataset	J Clin Oncol 2011 Jun 20;29(18):2582-9
469	Exploratory Dataset	Diabetes Obes Metab 2011 Jan;13(1):65-74
470	Exploratory Dataset	Diabet Med 2011 Nov;28(11):1352-61
471	Exploratory Dataset	Ann Rheum Dis, 2011, 70: 982-9
472	Exploratory Dataset	BMC Cancer 2012 Sep 24;12:423
473	Exploratory Dataset	Circulation, 2013, 127: 96-103
474	Exploratory Dataset	Arthritis Rheum 2012 Mar;64(3):876-84
475	Exploratory Dataset	Intensive Care Med, 2012, 38: 1118-25
476	Exploratory Dataset	Cancer 2017 Jan 1;123(2):303-311

© 2024 Zhang F et al. *JAMA Network Open*.

477	Exploratory Dataset	Cancer 2014 Jun 15;120(12):1838-46
478	Exploratory Dataset	Int J Clin Pract, 2011, 65: 1230-9
479	Exploratory Dataset	Clin Gastroenterol Hepatol 2014 Sep;12(9):1485-93e2
480	Exploratory Dataset	Diabetes Obes Metab 2011 Mar;13(3):258-67
481	Exploratory Dataset	JAMA 2014 Jan 8;311(2):145-54
482	Exploratory Dataset	J Diabetes Investig 2014 Jul;5(4):382-91
483	Exploratory Dataset	Lancet 2012 Aug 4;380(9840):475-83
484	Exploratory Dataset	BMC Pulm Med 2010 Mar 8;10:11
485	Exploratory Dataset	Diabetes Obes Metab 2013 Sep;15(9):810-8
486	Exploratory Dataset	J Clin Oncol 2012 Feb 1;30(4):372-9
487	Exploratory Dataset	N Engl J Med 2010 Feb 11;362(6):504-12
488	Exploratory Dataset	BMC Pulm Med 2011 Nov 15;11:51
489	Exploratory Dataset	Inflamm Bowel Dis 2010 Oct;16(10):1708-16
490	Exploratory Dataset	J Clin Oncol 2014 Mar 10;32(8):752-9
491	Exploratory Dataset	Clin Breast Cancer 2015 Feb;15(1):8-15
492	Exploratory Dataset	N Engl J Med 2016 Nov 10;375(19):1845-1855
493	Exploratory Dataset	Lancet Oncol, 2015, 16: 522-30
494	Exploratory Dataset	Am J Respir Crit Care Med 2014 Jul 15;190(2):208-17
495	Exploratory Dataset	Lancet 2010 Aug 7;376(9739):431-9
496	Exploratory Dataset	N Engl J Med, 2011, 364: 1995-2005
497	Exploratory Dataset	J Clin Oncol 2013 Jul 1;31(19):2477-84
498	Exploratory Dataset	Lancet Diabetes Endocrinol 2014 Jun;2(6):464-73
499	Exploratory Dataset	Diabetes Care, 2012, 35: 683-9
500	Exploratory Dataset	Diabetes Care 2012 Jun;35(6):1232-8

© 2024 Zhang F et al. *JAMA Network Open*.

501	Exploratory Dataset	N Engl J Med 2012 Dec 20;367(25):2385-95
502	Exploratory Dataset	Chin J Cancer, 2011, 30: 682-9
503	Exploratory Dataset	N Engl J Med 2013 Aug 29;369(9):799-808
504	Exploratory Dataset	Int J Clin Pract 2012 May;66(5):446-56
505	Exploratory Dataset	J Clin Psychiatry 2012 May;73(5):654-60
506	Exploratory Dataset	Cancer 2014 Sep 1;120(17):2684-93
507	Exploratory Dataset	Ann Allergy Asthma Immunol 2009 Jul;103(1):62-72
508	Exploratory Dataset	Allergy Asthma Proc Jan-Feb 2010;31(1):26-39
509	Exploratory Dataset	Current Medical Research and Opinion 2013;29(10):1357-69
510	Exploratory Dataset	Allergy Asthma Proc Jan-Feb 2010;31(1):49-59
511	Exploratory Dataset	Diabetes Obes Metab 2013 Apr;15(4):364-71
512	Exploratory Dataset	Clin Gastroenterol Hepatol 2008 Dec;6(12):1370-7
513	Exploratory Dataset	JAMA Oncol 2017 Nov 1;3(11):1520-1528
514	Exploratory Dataset	Lancet Oncol, 2012, 13: 528-38
515	Exploratory Dataset	Gut 2014 Mar;63(3):442-50
516	Exploratory Dataset	Thromb Haemost 2011 Apr;105(4):721-9
517	Exploratory Dataset	J Allergy Clin Immunol, 2013, 132: 1086-1096e5
518	Exploratory Dataset	N Engl J Med 2013 Aug 29;369(9):809-18
519	Exploratory Dataset	Diabetes Care 2011 Sep;34(9):2015-22
520	Exploratory Dataset	Diabetes Res Clin Pract 2011 Nov;94(2):217-24
521	Exploratory Dataset	Clin Genitourin Cancer 2012 Mar;10(1):6-14
522	Exploratory Dataset	Kidney Int 2014 Apr;85(4):962-71
523	Exploratory Dataset	Sci Transl Med 2010 Oct 6;2(52):52ra72
524	Exploratory Dataset	Diabetes Obes Metab 2014 Feb;16(2):124-36

© 2024 Zhang F et al. *JAMA Network Open*.

525	Exploratory Dataset	Ann Rheum Dis 2015 May;74(5):843-50
526	Exploratory Dataset	Diabetes Care 2012 Feb;35(2):252-8
527	Exploratory Dataset	J Clin Oncol 2014 Jan 10;32(2):76-82
528	Exploratory Dataset	Chest 2011 Jul;140(1):68-75
529	Exploratory Dataset	N Engl J Med 2012 Aug 23;367(8):716-24
530	Exploratory Dataset	Gut 2014 Mar;63(3):433-41
531	Exploratory Dataset	Gastroenterology 2012 Nov;143(5):1218-1226e2
532	Exploratory Dataset	N Engl J Med 2011 Oct 27;365(17):1586-96
533	Exploratory Dataset	Circulation 2014 Feb 18;129(7):764-72
534	Exploratory Dataset	Diabetes Ther 2014 Jun;5(1):267-83
535	Exploratory Dataset	PLoS One 2012;7(1):e30123
536	Exploratory Dataset	Diabetes Care 2012 Jul;35(7):1473-8
537	Exploratory Dataset	Clin Ther, 2013, 35: 431-9
538	Exploratory Dataset	Mod Rheumatol, 2015, 25: 514-21
539	Exploratory Dataset	Nicotine Tob Res 2012 Mar;14(3):343-50
540	Exploratory Dataset	Br J Dermatol, 2011, 165: 652-60
541	Exploratory Dataset	Lancet Oncol 2015 Oct;16(13):1344-54
542	Exploratory Dataset	J Thorac Oncol 2017 May;12(5):843-849
543	Exploratory Dataset	Schizophr Res 2014;152(2–3):450–7
544	Exploratory Dataset	Gut 2013 Aug;62(8):1122-30
545	Exploratory Dataset	Diabetes Metab Res Rev 2012 Mar;28(3):268-75
546	Exploratory Dataset	Lancet Oncol, 2013, 14: 29-37
547	Exploratory Dataset	Int J Clin Pract, 2011, 65: 397-407
548	Exploratory Dataset	Diabetes Obes Metab 2011 Feb;13(2):160-8

© 2024 Zhang F et al. *JAMA Network Open*.

549	Exploratory Dataset	J Asthma 2012 Feb;49(1):70-7
550	Exploratory Dataset	J Am Coll Cardiol, 2013, 62: 909-17
551	Exploratory Dataset	J Clin Oncol 2015 Jan 10;33(2):141-8
552	Exploratory Dataset	J Clin Oncol 2013 Oct 10;31(29):3639-46
553	Exploratory Dataset	Br J Psychiatry 2014 Aug;205(2):135-44
554	Exploratory Dataset	Br J Dermatol, 2011, 165: 661-8
555	Exploratory Dataset	J Diabetes Complications May-Jun 2014;28(3):386-92
556	Exploratory Dataset	J Clin Oncol, 2015, 33: 433-41
557	Exploratory Dataset	Rheumatology (Oxford) 2013 Jul;52(7):1303-12
558	Exploratory Dataset	BMJ 2010 Dec 6;341:c6549
559	Exploratory Dataset	Rheumatology (Oxford) 2012 Dec;51(12):2204-14
560	Exploratory Dataset	Ann Rheum Dis 2014 Sep;73(9):1616-25
561	Exploratory Dataset	Lancet Oncol 2014 Aug;15(9):1007-18
562	Exploratory Dataset	Breast Cancer Res Treat 2020 Sep;183(2):419-428
563	Exploratory Dataset	Cancer Chemother Pharmacol 2015 Mar;75(3):569-77
564	Exploratory Dataset	Diabetes Obes Metab 2014 Mar;16(3):223-30
565	Exploratory Dataset	J Clin Psychopharmacol 2013 Apr;33(2):243-7
566	Exploratory Dataset	Ann Rheum Dis 2012 Jun;71(6):817-24
567	Exploratory Dataset	Diabetes Obes Metab, 2015, 17: 849-58
568	Exploratory Dataset	J Am Acad Child Adolesc Psychiatry 2011 Apr;50(4):395-405
569	Exploratory Dataset	Gut 2015 Aug;64(8):1227-35
570	Exploratory Dataset	Diabetes Obes Metab 2012 Dec;14(12):1145-54
571	Exploratory Dataset	Aliment Pharmacol Ther 2010 Oct;32(8):990-9
572	Exploratory Dataset	Diabetes Obes Metab 2013 Dec;15(12):1154-60

© 2024 Zhang F et al. *JAMA Network Open*.

573	Exploratory Dataset	Biol Psychiatry 2011 Jun 1;69(11):1075-82
574	Exploratory Dataset	Lancet 2012 Jun 16;379(9833):2262-9
575	Exploratory Dataset	Eur J Cancer 2015 Mar;51(4):522-532
576	Exploratory Dataset	Lancet Oncol 2014 Jul;15(8):852-61
577	Exploratory Dataset	Ann Oncol 2015 Mar;26(3):542-7
578	Exploratory Dataset	Jpn J Clin Oncol 2016 Mar;46(3):248-53
579	Exploratory Dataset	Lancet 2012 May 19;379(9829):1879-86
580	Exploratory Dataset	Clin Drug Investig 2013 Oct;33(10):707-17
581	Exploratory Dataset	Curr Med Res Opin 2012 Apr;28(4):513-23
582	Exploratory Dataset	Diabetes Obes Metab 2013 Nov;15(11):1000-7
583	Exploratory Dataset	Eur Neuropsychopharmacol 2013 Oct;23(10):1208-18
584	Exploratory Dataset	Ann Intern Med 2011 Jan 18;154(2):103-12
585	Exploratory Dataset	Am J Emerg Med, 2015, 33: 14-20
586	Exploratory Dataset	Ann Intern Med 2013 May 7;158(9):641-9
587	Exploratory Dataset	BMC Cancer 2014 Apr 25;14:290
588	Exploratory Dataset	Lancet Oncol, 2012, 13: 466-75
589	Exploratory Dataset	N Engl J Med 2012 Oct 18;367(16):1519-28
590	Exploratory Dataset	Eur J Cancer 2013 Dec;49(18):3763-72
591	Exploratory Dataset	J Clin Oncol 2013 Sep 10;31(26):3212-8
592	Exploratory Dataset	Lung Cancer 2013 Dec;82(3):455-60
593	Exploratory Dataset	J Clin Oncol, 2011, 29: 3307-15
594	Exploratory Dataset	N Engl J Med 2013 Nov 28;369(22):2093-104
595	Exploratory Dataset	Lipids Health Dis 2012 Jan 31;11:18
596	Exploratory Dataset	Am J Cardiol 2011 Aug 15;108(4):523-30

© 2024 Zhang F et al. *JAMA Network Open*.

597	Exploratory Dataset	N Engl J Med 2013; 369:711-721
598	Exploratory Dataset	N Engl J Med 2013 Aug 22;369(8):699-710
599	Exploratory Dataset	BMC Musculoskelet Disord 2011 Jul 7;12:153
600	Exploratory Dataset	N Engl J Med 2012; 367:616-624
601	Exploratory Dataset	Clin Cancer Res 2014 Aug 15;20(16):4240-50
602	Exploratory Dataset	Diabetes Obes Metab 2013 Aug;15(8):721-8
603	Exploratory Dataset	Clin Ther 2012 Mar;34(3):580-92
604	Exploratory Dataset	Lancet 2013 Jan 12;381(9861):125-32
605	Exploratory Dataset	N Engl J Med 2015 Jul 16;373(3):232-42
606	Exploratory Dataset	Lancet 2013 Mar 9;381(9869):817-24
607	Exploratory Dataset	Drug Alcohol Depend 2013 Dec 1;133(2):486-93
608	Exploratory Dataset	Gynecol Oncol 2013 Jul;130(1):25-30
609	Exploratory Dataset	Mod Rheumatol 2014 Jul;24(4):552-60
610	Exploratory Dataset	Mod Rheumatol 2014 Sep;24(5):715-24
611	Exploratory Dataset	Int J Chron Obstruct Pulmon Dis 2014 Jul 5;9:697-714
612	Exploratory Dataset	Eur J Cancer 2014 Mar;50(4):706-12
613	Exploratory Dataset	Diabetes Obes Metab 2012 Jun;14(6):565-74
614	Exploratory Dataset	Diabetes Obes Metab 2015 Oct;17(10):984-93
615	Exploratory Dataset	N Engl J Med, 2014, 370: 734-43
616	Exploratory Dataset	Ann Oncol 2015 Oct;26(10):2173-9
617	Exploratory Dataset	Lancet Oncol 2014 Feb;15(2):143-55
618	Exploratory Dataset	Exp Dermatol 2015 Jul;24(7):529-35
619	Exploratory Dataset	Lancet 2013 Nov 23;382(9906):1705-13
620	Exploratory Dataset	Ann Rheum Dis 2014 Feb;73(2):349-56

© 2024 Zhang F et al. *JAMA Network Open*.

621	Exploratory Dataset	Ann Rheum Dis 2013 Jan;72(1):43-50
622	Exploratory Dataset	Biol Psychiatry 2013 Apr 15;73(8):706-13
623	Exploratory Dataset	Alcohol Alcohol Sep-Oct 2013;48(5):570-8
624	Exploratory Dataset	J Diabetes 2012 Sep;4(3):227-37
625	Exploratory Dataset	Neurology 2016 Jul 5;87(1):57-64
626	Exploratory Dataset	N Engl J Med, 2012, 367: 495-507
627	Exploratory Dataset	Clin Ther 2012 Sep;34(9):1909-19e15
628	Exploratory Dataset	J Clin Oncol 2014 Jul 20;32(21):2240-7
629	Exploratory Dataset	J Clin Oncol 2010 Sep 1;28(25):3945-50
630	Exploratory Dataset	Lancet Oncol 2013 May;14(6):481-9
631	Exploratory Dataset	J Clin Oncol, 2013, 31: 3509-16
632	Exploratory Dataset	JAMA Surg, 2015, 150: 730-7
633	Exploratory Dataset	J Clin Oncol, 2014, 32: 2463-70
634	Exploratory Dataset	N Engl J Med 2012; 367:1783-1791
635	Exploratory Dataset	Arthritis Care Res (Hoboken) 2012 Oct;64(10):1462-70
636	Exploratory Dataset	JAMA Psychiatry 2015 May;72(5):430-7
637	Exploratory Dataset	N Engl J Med 2011 Aug 25;365(8):699-708
638	Exploratory Dataset	Lancet Oncol 2013 Nov;14(12):1233-42
639	Exploratory Dataset	Diabetes Care 2014 Aug;37(8):2141-8
640	Exploratory Dataset	Diabetologia 2014 Dec;57(12):2475-84
641	Exploratory Dataset	Diabetes Obes Metab 2015 Feb;17(2):179-87
642	Exploratory Dataset	Ann Rheum Dis 2014 Jan;73(1):101-7
643	Exploratory Dataset	Arthritis Rheum 2013 Mar;65(3):559-70
644	Exploratory Dataset	Diabetologia, 2016, 59: 266-74

© 2024 Zhang F et al. *JAMA Network Open*.

645	Exploratory Dataset	Diabetes Obes Metab 2014 Dec;16(12):1257-64
646	Exploratory Dataset	N Engl J Med 2012 Aug 9;367(6):508-19
647	Exploratory Dataset	J Clin Oncol 2013 Nov 10;31(32):4105-14
648	Exploratory Dataset	Diabetes Obes Metab 2014 Feb;16(2):159-69
649	Exploratory Dataset	J Hepatol 2016 May;64(5):1090-1098
650	Exploratory Dataset	J Diabetes Complications, 2015, 29: 1287-94
651	Exploratory Dataset	J Rheumatol 2014 Aug;41(8):1703-11
652	Exploratory Dataset	Diabetes Obes Metab 2014 Dec;16(12):1239-46
653	Exploratory Dataset	Ann Intern Med 2013 Aug 20;159(4):253-61
654	Exploratory Dataset	Lancet Oncol 2014 Jun;15(7):700-12
655	Exploratory Dataset	Clin Breast Cancer 2013 Dec;13(6):421-432e8
656	Exploratory Dataset	J Clin Oncol 2012 Aug 1;30(22):2718-24
657	Exploratory Dataset	Adv Ther 2013 Oct;30(10):870-84
658	Exploratory Dataset	N Engl J Med 2012 Feb 9;366(6):520-9
659	Exploratory Dataset	J Thorac Oncol 2015 Dec;10(12):1745-53
660	Exploratory Dataset	Clin Cancer Res 2013 May 1;19(9):2541-50
661	Exploratory Dataset	J Clin Oncol 2014 Oct 20;32(30):3374-82
662	Exploratory Dataset	Ann Rheum Dis 2014 Mar;73(3):536-43
663	Exploratory Dataset	Lancet Oncol 2015 Jul;16(7):816-29
664	Exploratory Dataset	Blood 2014 May 8;123(19):2944-52
665	Exploratory Dataset	Lancet Oncol, 2016, 17: 367-377
666	Exploratory Dataset	J Clin Oncol 2013 Nov 1;31(31):3935-43
667	Exploratory Dataset	Diabetes Care, 2013, 36: 4015-21
668	Exploratory Dataset	N Engl J Med, 2014, 370: 699-708

© 2024 Zhang F et al. *JAMA Network Open*.

669	Exploratory Dataset	J Diabetes Complications Mar-Apr 2013;27(2):177-83
670	Exploratory Dataset	N Engl J Med, 2013, 368: 138-48
671	Exploratory Dataset	Gastric Cancer, 2015, 18: 168-76
672	Exploratory Dataset	Lancet Oncol, 2013, 14: 38-47
673	Exploratory Dataset	Lancet Oncol 2015 Dec;16(16):1691-9
674	Exploratory Dataset	Eur Respir J 2013 Dec;42(6):1622-32
675	Exploratory Dataset	J Child Adolesc Psychopharmacol 2013 Feb;23(1):3-10
676	Exploratory Dataset	Hepatology, 2014, 60: 1697-707
677	Exploratory Dataset	Lancet, 2014, 383: 31-39
678	Exploratory Dataset	Respir Med 2008 May;102(5):665-73
679	Exploratory Dataset	Alcohol Clin Exp Res 2018 Apr;42(4):751-760
680	Exploratory Dataset	J Child Adolesc Psychopharmacol 2014 May;24(4):190-200
681	Exploratory Dataset	Ann Rheum Dis 2013 Jun;72(6):863-9
682	Exploratory Dataset	J Rheumatol 2014 Mar;41(3):414-21
683	Exploratory Dataset	Gut 2011 Aug;60(8):1068-75
684	Exploratory Dataset	J Psychiatr Res, 2012, 46: 574-81
685	Exploratory Dataset	Clin Ther 2012 Sep;34(9):1892-908e1
686	Exploratory Dataset	Lancet Oncol, 2013, 14: 733-40
687	Exploratory Dataset	Alcohol Clin Exp Res 2017 Jun;41(6):1201-1211
688	Exploratory Dataset	Ann Rheum Dis 2013 Jun;72(6):815-22
689	Exploratory Dataset	Lancet Oncol 2015 Aug;16(8):967-78
690	Exploratory Dataset	Br J Dermatol 2013 Feb;168(2):402-11
691	Exploratory Dataset	Thromb Res 2012 Sep;130(3):e52-9
692	Exploratory Dataset	N Engl J Med, 2014, 370: 709-22

© 2024 Zhang F et al. *JAMA Network Open*.

693	Exploratory Dataset	J Clin Oncol, 2017, 35: 48-55
694	Exploratory Dataset	J Rheumatol 2015 Jun;42(6):912-9
695	Exploratory Dataset	AIDS 2013 Jul 17;27(11):1771-8
696	Exploratory Dataset	Diabetes Care 2013 Dec;36(12):3875-81
697	Exploratory Dataset	Rheumatology (Oxford) 2013 Jul;52(7):1285-92
698	Exploratory Dataset	Lancet 2013 Feb 9;381(9865):451-60
699	Exploratory Dataset	Diabetes Care 2014 Oct;37(10):2763-73
700	Exploratory Dataset	J Clin Oncol 2013 Aug 20;31(24):3004-11
701	Exploratory Dataset	Arthritis Rheumatol 2014 Jul;66(7):1693-704
702	Exploratory Dataset	Eur J Cancer 2013 Nov;49(17):3609-15
703	Exploratory Dataset	N Engl J Med 2013 Oct 3;369(14):1327-35
704	Exploratory Dataset	Diabetes Care, 2015, 38: 355-64
705	Exploratory Dataset	Lancet 2013 Sep 14;382(9896):941-50
706	Exploratory Dataset	Postgrad Med 2016 May;128(4):371-80
707	Exploratory Dataset	J Clin Oncol 2013 Sep 10;31(26):3219-25
708	Exploratory Dataset	Diabetes Obes Metab 2013 May;15(5):432-40
709	Exploratory Dataset	Ann Rheum Dis 2013 Mar;72(3):381-9
710	Exploratory Dataset	N Engl J Med, 2012, 367: 1187-97
711	Exploratory Dataset	Diabetes Care 2013 Sep;36(9):2489-96
712	Exploratory Dataset	N Engl J Med 2012 Mar 29;366(13):1181-9
713	Exploratory Dataset	Diabetes Care 2014 Aug;37(8):2317-25
714	Exploratory Dataset	J Diabetes Complications, 2017, 31: 1283-1285
715	Exploratory Dataset	Diabetes Obes Metab 2013 Sep;15(9):853-62
716	Exploratory Dataset	J Clin Oncol, 2014, 32: 1302-8

© 2024 Zhang F et al. *JAMA Network Open*.

717	Exploratory Dataset	Nicotine Tob Res 2013 Feb;15(2):419-27
718	Exploratory Dataset	Lancet 2014 Jul 26;384(9940):319-28
719	Exploratory Dataset	BMC Cancer 2011 Aug 11;11:349
720	Exploratory Dataset	Diabetes Care 2014;37(3):740-50
721	Exploratory Dataset	Lancet Respir Med, 2014, 2: 361-8
722	Exploratory Dataset	N Engl J Med 2013 Oct 10;369(15):1406-15
723	Exploratory Dataset	Ann Rheum Dis 2015 Jun;74(6):1110-7
724	Exploratory Dataset	Lancet Oncol 2013 Jan;14(1):55-63
725	Exploratory Dataset	Lancet 2013 Apr 6;381(9873):1203-10
726	Exploratory Dataset	Neurology 2012 Feb 21;78(8):545-50
727	Exploratory Dataset	J Diabetes 2017 Apr;9(4):412-422
728	Exploratory Dataset	J Rheumatol 2014 Apr;41(4):648-57
729	Exploratory Dataset	Lancet Oncol, 2014, 15: 1379-88
730	Exploratory Dataset	Malawi Med J 2016 Sep;28(3):123-130
731	Exploratory Dataset	Br J Dermatol 2012;167:649-57
732	Exploratory Dataset	Diabetes Obes Metab 2015 Jul;17(7):630-8
733	Exploratory Dataset	Ann Rheum Dis, 2016, 75: 1081-91
734	Exploratory Dataset	Ann Rheum Dis 2017 Jul;76(7):1279-1284
735	Exploratory Dataset	Lancet Oncol 2014 May;15(6):580-91
736	Exploratory Dataset	Gastroenterology 2015 Apr;148(4):740-750e2
737	Exploratory Dataset	PLoS One, 2017, 12: e0182411
738	Exploratory Dataset	Lancet 2013 Aug 31;382(9894):780-9
739	Exploratory Dataset	Mol Cancer 2010 Mar 30;9:69
740	Exploratory Dataset	Gut 2012 Dec;61(12):1693-700

© 2024 Zhang F et al. *JAMA Network Open*.

741	Exploratory Dataset	Eur Respir J, 2011, 37: 164-72
742	Exploratory Dataset	J Clin Psychiatry 2014 Jul;75(7):765-72
743	Exploratory Dataset	Diabetes Obes Metab 2015 Oct;17(10):936-48
744	Exploratory Dataset	Psychopharmacology (Berl) 2012 Oct;223(3):299-306
745	Exploratory Dataset	Lancet Oncol, 2016, 17: 78-89
746	Exploratory Dataset	BMC Cancer 2016 Aug 31;16(1):699
747	Exploratory Dataset	Diabetes Obes Metab 2013 Dec;15(12):1136-45
748	Exploratory Dataset	Diabetes Obes Metab 2014 Jul;16(7):613-21
749	Exploratory Dataset	Diabetes Res Clin Pract 2014 Oct;106(1):50-6
750	Exploratory Dataset	Addiction 2014 Sep;109(9):1554-63
751	Exploratory Dataset	Diabetes Care 2015 Jul;38(7):1218-27
752	Exploratory Dataset	PLoS One 2013 Nov 11;8(11):e62264
753	Exploratory Dataset	Diabetes Care 2015 Mar;38(3):403-11
754	Exploratory Dataset	N Engl J Med, 2017, 377: 644-657
755	Exploratory Dataset	Lancet, 2016, 388: 343-355
756	Exploratory Dataset	JAMA 2014 Jul 2;312(1):57-67
757	Exploratory Dataset	N Engl J Med 2016 Jul 7;375(1):23-34
758	Exploratory Dataset	J Am Geriatr Soc 2014 Jul;62(7):1252-62
759	Exploratory Dataset	Diabetes Obes Metab 2013 Aug;15(8):760-6
760	Exploratory Dataset	Arthritis Rheumatol 2015 Feb;67(2):334-43
761	Exploratory Dataset	Respir Med 2013 Apr;107(4):560-9
762	Exploratory Dataset	Diabetology International 2015; 6(1): 8-18
763	Exploratory Dataset	J Clin Oncol 2017 Jan;35(1):40-47
764	Exploratory Dataset	J Affect Disord 2015;174:296–302

© 2024 Zhang F et al. *JAMA Network Open*.

765	Exploratory Dataset	J Clin Psychiatry 2015;76(3):284–92
766	Exploratory Dataset	Gastroenterology 2013 Jul;145(1):149-157e2
767	Exploratory Dataset	Diabetes Obes Metab 2015 Jun;17(6):591-598
768	Exploratory Dataset	Ann Rheum Dis 2015 Jun;74(6):1051-7
769	Exploratory Dataset	Arthritis Rheumatol 2015 Jun;67(6):1424-37
770	Exploratory Dataset	Ann Rheum Dis 2014 Sep;73(9):1626-34
771	Exploratory Dataset	Ann Rheum Dis, 2014, 73: 376-84
772	Exploratory Dataset	J Clin Oncol 2015 Nov 20;33(33):3858-65
773	Exploratory Dataset	Diabetes Obes Metab 2014 Oct;16(10):1016-27
774	Exploratory Dataset	Clin Colorectal Cancer 2015 Sep;14(3):162-9
775	Exploratory Dataset	Respirology 2013 Jul;18(5):866-73
776	Exploratory Dataset	Respir Res, 2013, 14: 125
777	Exploratory Dataset	Br J Dermatol 2013 Feb;168(2):412-21
778	Exploratory Dataset	J Diabetes Complications May-Jun 2013;27(3):268-73
779	Exploratory Dataset	N Engl J Med 2012 Nov 1;367(18):1694-703
780	Exploratory Dataset	Int J Cancer 2016 Jul 1;139(1):177-86
781	Exploratory Dataset	Ann Rheum Dis 2014 Jun;73(6):990-9
782	Exploratory Dataset	Ann Intern Med 2013 Sep 17;159(6):390-400
783	Exploratory Dataset	Curr Med Res Opin 2014 Feb;30(2):163-75
784	Exploratory Dataset	Diabetes Obes Metab 2013 Apr;15(4):372-82
785	Exploratory Dataset	Lancet Oncol 2015 Jan;16(1):87-97
786	Exploratory Dataset	Diabetes Care 2015 Feb;38(2):e15-7
787	Exploratory Dataset	Ann Rheum Dis 2014 Jan;73(1):39-47
788	Exploratory Dataset	Ann Rheum Dis 2014 Jan;73(1):48-55

© 2024 Zhang F et al. *JAMA Network Open*.

789	Exploratory Dataset	Clin Lung Cancer 2015 Nov;16(6):447-56
790	Exploratory Dataset	Clin Ther 2014 Jan 1;36(1):84-100e9
791	Exploratory Dataset	J Diabetes 2016 Nov;8(6):796-808
792	Exploratory Dataset	Diabetes Obes Metab 2015 Aug;17(8):805-8
793	Exploratory Dataset	Diabetes Care, 2014, 37: 2723-30
794	Exploratory Dataset	Diabetes Res Clin Pract 2015 Jul;109(1):141-8
795	Exploratory Dataset	Lancet 2013 Jan 26;381(9863):303-12
796	Exploratory Dataset	J Clin Psychiatry 2015;76(12):e1574–82
797	Exploratory Dataset	J Clin Psychopharmacol 2015;35(4):367–73
798	Exploratory Dataset	Int J Clin Pract 2013 Dec;67(12):1267-82
799	Exploratory Dataset	Diabetes Obes Metab 2015 Mar;17(3):294-303
800	Exploratory Dataset	Hosp Pract (1995), 2013, 41: 72-84
801	Exploratory Dataset	Diabetologia 2013 Dec;56(12):2582-92
802	Exploratory Dataset	Diabetes Obes Metab 2014 May;16(5):467-77
803	Exploratory Dataset	N Engl J Med 2012 Mar 29;366(13):1190-9
804	Exploratory Dataset	N Engl J Med 2013 Oct 3;369(14):1317-26
805	Exploratory Dataset	Ann Rheum Dis 2014 Mar;73(3):587-94
806	Exploratory Dataset	Diabetes Obes Metab 2013 May;15(5):403-9
807	Exploratory Dataset	Lancet, 2013, 381: 1541-50
808	Exploratory Dataset	Diabetes Care, 2014, 37: 2168-76
809	Exploratory Dataset	N Engl J Med 2015 Nov 26;373(22):2117-28
810	Exploratory Dataset	Diabetes Obes Metab 2015 Mar;17(3):304-8
811	Exploratory Dataset	Blood Press 2016;25(2):93-103
812	Exploratory Dataset	Diabetes Care 2013 Sep;36(9):2508-15

© 2024 Zhang F et al. *JAMA Network Open*.

813	Exploratory Dataset	Lancet Oncol, 2015, 16: 859-70
814	Exploratory Dataset	JAMA, 2014, 311: 2397-405
815	Exploratory Dataset	N Engl J Med, 2017, 377: 1228-1239
816	Exploratory Dataset	J Addict Med Jul-Aug 2013;7(4):277-86
817	Exploratory Dataset	N Engl J Med 2015 Dec 3;373(23):2247-57 (作者仅给出非学术网页链接，我们根据 Trial 名称找到对应文献)
818	Exploratory Dataset	J Intern Med, 2015, 277: 137-50
819	Exploratory Dataset	Cancer Med 2015 Jan;4(1):16-26
820	Exploratory Dataset	Diabet Med 2015 Dec;32(12):1555-67
821	Exploratory Dataset	Eur J Cancer, 2015, 51: 1212-20
822	Exploratory Dataset	Cancer 2015 Mar 15;121(6):883-92
823	Exploratory Dataset	Ann Rheum Dis, 2015, 74: 818-22
824	Exploratory Dataset	Lancet Diabetes Endocrinol 2014 May;2(5):369-84
825	Exploratory Dataset	Diabetes Obes Metab, 2018, 20: 2768-2777
826	Exploratory Dataset	Lancet Diabetes Endocrinol 2014 Sep;2(9):691-700
827	Exploratory Dataset	Lancet 2014 Aug 23;384(9944):665-73
828	Exploratory Dataset	Diabetes Metab Res Rev, 2014, 30: 726-35
829	Exploratory Dataset	Lancet Oncol 2014 Oct;15(11):1224-35
830	Exploratory Dataset	Lancet Diabetes Endocrinol 2013 Nov;1(3):208-19
831	Exploratory Dataset	N Engl J Med, 2016, 375: 311-22
832	Exploratory Dataset	Diabetes Obes Metab 2017 Aug;19(8):1188-1192
833	Exploratory Dataset	Lancet Oncol 2015 May;16(5):499-508
834	Exploratory Dataset	Ann Rheum Dis 2015 Feb;74(2):333-40
835	Exploratory Dataset	Ann Oncol 2015 Sep;26(9):1904-1910

© 2024 Zhang F et al. *JAMA Network Open*.

836	Exploratory Dataset	Dermatol Surg, 2015, 41: 102-12
837	Exploratory Dataset	Drugs Aging 2015 Jun;32(6):469-76
838	Exploratory Dataset	Adv Ther 2014 Jun;31(6):621-38
839	Exploratory Dataset	Lancet Oncol, 2015, 16: 338-48
840	Exploratory Dataset	J Clin Oncol, 2015, 33: 723-31
841	Exploratory Dataset	Expert Opin Pharmacother 2012 Dec;13(17):2443-52
842	Exploratory Dataset	Lancet Diabetes Endocrinol 2016 Mar;4(3):211-220
843	Exploratory Dataset	Eur Respir J 2013 Dec;42(6):1484-94
844	Exploratory Dataset	Diabetes Obes Metab 2013 Sep;15(9):833-43
845	Exploratory Dataset	Lancet Oncol, 2014, 15: 799-808
846	Exploratory Dataset	J Clin Oncol, 2012, 30: 362-71
847	Exploratory Dataset	J Pain, 2014, 15: 835-44
848	Exploratory Dataset	Eur J Cancer 2015 Aug;51(12):1511-28
849	Exploratory Dataset	Clin Ther 2015 Aug;37(8):1773-88e1
850	Exploratory Dataset	Ann Oncol, 2015, 26: 2457-63
851	Exploratory Dataset	Ann Emerg Med 2012 Jun;59(6):504-12e1-2
852	Exploratory Dataset	N Engl J Med, 2014, 371: 424-33
853	Exploratory Dataset	J Clin Oncol 2016 Feb 10;34(5):427-35
854	Exploratory Dataset	Diabetes Obes Metab 2015 Jan;17(1):42-51
855	Exploratory Dataset	J Antimicrob Chemother, 2014, 69: 1111-8
856	Exploratory Dataset	Gastroenterology 2014 Sep;147(3):618-627e3
857	Exploratory Dataset	Lancet Infect Dis 2013 Nov;13(11):927-35
858	Exploratory Dataset	Lancet 2013 Aug 24;382(9893):700-8
859	Exploratory Dataset	Eur J Cancer, 2014, 50: 2072-81

© 2024 Zhang F et al. *JAMA Network Open*.

860	Exploratory Dataset	Arthritis Care Res (Hoboken), 2014, 66: 1653-61
861	Exploratory Dataset	J Clin Oncol 2012 May 1;30(13):1484-91
862	Exploratory Dataset	N Engl J Med, 2016, 375: 1231-41
863	Exploratory Dataset	Br J Cancer 2012 Apr 24;106(9):1469-74
864	Exploratory Dataset	Int Arch Allergy Immunol, 2016, 169: 135-45
865	Exploratory Dataset	Lancet Respir Med, 2014, 2: 879-890
866	Exploratory Dataset	Lancet, 2015, 386: 552-61
867	Exploratory Dataset	Diabetol Int 6, 125–138 (2015)
868	Exploratory Dataset	Ann Rheum Dis 2014 Sep;73(9):1607-15
869	Exploratory Dataset	Clin Pharmacol Ther 2014 Sep;96(3):390-6
870	Exploratory Dataset	J Clin Oncol 2015 Aug 20;33(24):2667-74
871	Exploratory Dataset	N Engl J Med, 2012, 367: 107-14
872	Exploratory Dataset	Contemp Clin Trials 2017 Sep;60:72-77
873	Exploratory Dataset	Ann Oncol 2016 Dec;27(12):2196-2203
874	Exploratory Dataset	Ann Oncol, 2014, 25: 968-74
875	Exploratory Dataset	Korean J Intern Med 2019 Jul;34(4):917-931
876	Exploratory Dataset	Lancet 2013 Aug 3;382(9890):409-416
877	Exploratory Dataset	Arthritis Rheumatol 2014 Aug;66(8):2091-102
878	Exploratory Dataset	Ann Emerg Med 2015 May;65(5):479-488e2
879	Exploratory Dataset	Epilepsia, 2015, 56: 1890-8
880	Exploratory Dataset	J Acquir Immune Defic Syndr 2015 Dec 15;70(5):515-9
881	Exploratory Dataset	Ann Oncol 2015 May;26(5):943-949
882	Exploratory Dataset	J Clin Endocrinol Metab 2016 Jun;101(6):2528-35
883	Exploratory Dataset	Neurotherapeutics 2015 Apr;12(2):455-60

© 2024 Zhang F et al. *JAMA Network Open*.

884	Exploratory Dataset	Lancet 2013 Jan 26;381(9863):295-302
885	Exploratory Dataset	Arthritis Rheumatol 2016 Sep;68(9):2174-83
886	Exploratory Dataset	J Am Acad Dermatol 2016 May;74(5):841-50
887	Exploratory Dataset	Aliment Pharmacol Ther 2015 Nov;42(10):1170-81
888	Exploratory Dataset	Gut 2015 Jun;64(6):894-900
889	Exploratory Dataset	J Clin Oncol, 2017, 35: 3449-3457
890	Exploratory Dataset	Lancet Oncol, 2016, 17: 153-163
891	Exploratory Dataset	Diabetes Res Clin Pract 2015 Oct;110(1):82-90
892	Exploratory Dataset	Cardiovasc Diabetol 2015 Dec 23;14:154
893	Exploratory Dataset	J Crohns Colitis 2016 Apr;10(4):418-28
894	Exploratory Dataset	JAMA Psychiatry 2016 Oct 1;73(10):1056-1063
895	Exploratory Dataset	Clin Cancer Res 2015 Apr 1;21(7):1574-82
896	Exploratory Dataset	Diabetes Care 2014 Jul;37(7):1815-23
897	Exploratory Dataset	Respir Med 2013 Oct;107(10):1538-46
898	Exploratory Dataset	Diabetes Obes Metab 2015 Feb;17(2):152-60
899	Exploratory Dataset	Clin Ther 2015 Jul 1;37(7):1493-502
900	Exploratory Dataset	J Diabetes Investig 2012 Dec 20;3(6):517-25
901	Exploratory Dataset	Anesthesiology<br>2012 Oct;117(4):755-64
902	Exploratory Dataset	N Engl J Med 2015; 372:621-630
903	Exploratory Dataset	Ther Adv Neurol Disord 2017 Sep;10(9):315-325
904	Exploratory Dataset	Pediatrics, 2011, 128: e1496-501
905	Exploratory Dataset	Lancet 2014 Jul 26;384(9940):309-18
906	Exploratory Dataset	J Clin Transl Endocrinol 2014 May 5;1(2):54-60

© 2024 Zhang F et al. *JAMA Network Open*.

907	Exploratory Dataset	Arthritis Rheumatol 2017 Jun;69(6):1144-1153
908	Exploratory Dataset	N Engl J Med 2015 Dec 24;373(26):2534-48
909	Exploratory Dataset	J Rheumatol 2016 Mar;43(3):495-503
910	Exploratory Dataset	Respiratory Medicine 2015;109(2):170-9
911	Exploratory Dataset	Ann Oncol, 2015, 26: 894-901
912	Exploratory Dataset	N Engl J Med 2014 Jul 24;371(4):326-38
913	Exploratory Dataset	Diabetes Obes Metab, 2015, 17: 665-74
914	Exploratory Dataset	N Engl J Med 2016 Nov 17;375(20):1946-1960
915	Exploratory Dataset	Diabetes Care 2015 Mar;38(3):420-8
916	Exploratory Dataset	JAMA 2015 Feb 17;313(7):687-94
917	Exploratory Dataset	Arthritis Rheumatol 2015 Oct;67(10):2591-600
918	Exploratory Dataset	Acta Oncol 2012 Sep;51(7):860-6
919	Exploratory Dataset	Diabetes Care 2015 Mar;38(3):431-8
920	Exploratory Dataset	Rheumatol Ther 2017 Dec;4(2):475-488
921	Exploratory Dataset	Diabetes Obes Metab 2015 Jan;17(1):23-31
922	Exploratory Dataset	Cancer 2016 Feb 15;122(4):574-81
923	Exploratory Dataset	N Engl J Med 2015 Oct;373(14):1329-39
924	Exploratory Dataset	Diabetes Obes Metab 2015 Nov;17(11):1075-84
925	Exploratory Dataset	Gut 2017 Jun;66(6):1049-1059
926	Exploratory Dataset	Br J Cancer 2016 Nov 8;115(10):1206-1214
927	Exploratory Dataset	Expert Opin Pharmacother 2014 Aug;15(11):1501-15
928	Exploratory Dataset	Oncologist 2017 Mar;22(3):264-271
929	Exploratory Dataset	Diabetes Care, 2015, 38: 384-93
930	Exploratory Dataset	Diabetes Care 2015 Mar;38(3):394-402

© 2024 Zhang F et al. *JAMA Network Open*.

931	Exploratory Dataset	Ann Hematol 2013 May;92(5):653-60
932	Exploratory Dataset	Rheumatology (Oxford) 2016 Jan;55(1):49-55
933	Exploratory Dataset	N Engl J Med 2015 Apr 16;372(16):1500-9
934	Exploratory Dataset	N Engl J Med 2016 Sep 1;375(9):850-60
935	Exploratory Dataset	JAMA Intern Med 2016 Nov 1;176(11):1630-1637
936	Exploratory Dataset	Lancet 2014 Jun 28;383(9936):2222-31
937	Exploratory Dataset	Lancet 2016 May 7;387(10031):1921-7
938	Exploratory Dataset	J Clin Oncol 2016 Nov 1;34(31):3740-3748
939	Exploratory Dataset	J Clin Oncol 2017 Feb;35(4):412-420
940	Exploratory Dataset	Lancet Oncol 2016 Jan;17(1):57-66
941	Exploratory Dataset	Diabetes Ther 2015 Jun;6(2):127-42
942	Exploratory Dataset	N Engl J Med 2016 Sep 1;375(9):840-9
943	Exploratory Dataset	BMC Pulm Med 2014 Nov 18;14:178
944	Exploratory Dataset	J Crohns Colitis 2017 Jul 1;11(7):811-819
945	Exploratory Dataset	J Rheumatol 2016 Mar;43(3):504-11
946	Exploratory Dataset	J Clin Psychiatry 2016;77(3):371–8
947	Exploratory Dataset	J Allergy Clin Immunol 2019 Apr;143(4):1395-1402
948	Exploratory Dataset	N Engl J Med 2016 Jul 28;375(4):345-56
949	Exploratory Dataset	N Engl J Med 2016 May 12;374(19):1822-30
950	Exploratory Dataset	Gut 2015 Feb;64(2):243-9
951	Exploratory Dataset	N Engl J Med, 2015, 373: 136-44
952	Exploratory Dataset	J Allergy Clin Immunol 2015 May;135(5):1171-8e1
953	Exploratory Dataset	Arthritis Rheumatol 2016 Sep;68(9):2163-73
954	Exploratory Dataset	J Crohns Colitis 2016 Nov;10(11):1294-1302

© 2024 Zhang F et al. *JAMA Network Open*.

955	Exploratory Dataset	Pain Physician Nov-Dec 2013;16(6):E749-62
956	Exploratory Dataset	N Engl J Med 2013 May 16;368(20):1878-87
957	Exploratory Dataset	J Diabetes Investig 2016 May;7(3):366-73
958	Exploratory Dataset	Ann Oncol 2018 Jan; 29(1): 154–161
959	Exploratory Dataset	N Engl J Med 2015 Apr 16;372(16):1489-99
960	Exploratory Dataset	N Engl J Med 2014 May 8;370(19):1809-19
961	Exploratory Dataset	N Engl J Med 2014 Jun 12;370(24):2295-306
962	Exploratory Dataset	J Crohns Colitis 2017 Jul 1;11(7):785-791
963	Exploratory Dataset	Ann Rheum Dis 2018 Feb;77(2):212-220
964	Exploratory Dataset	Eur J Anaesthesiol 2014 Dec;31(12):654-62
965	Exploratory Dataset	J Neurol Sci 2013 Jul 15;330(1-2):94-9
966	Exploratory Dataset	Lancet 2016 Sep 24;388(10051):1281-90
967	Exploratory Dataset	N Engl J Med 2013 May 16;368(20):1867-77
968	Exploratory Dataset	Diabetes Metab Res Rev 2014 Oct;30(7):582-9
969	Exploratory Dataset	Arthritis Rheumatol 2017 Apr;69(4):709-719
970	Exploratory Dataset	Br J Dermatol 2015 Feb;172(2):484-93
971	Exploratory Dataset	Diabetes Obes Metab, 2015, 17: 974-83
972	Exploratory Dataset	Clin Cancer Res 2017 Apr 15;23(8):1937-1944
973	Exploratory Dataset	J Clin Oncol 2013 Jun 1;31(16):1931-8
974	Exploratory Dataset	N Engl J Med, 2014, 371: 213-23
975	Exploratory Dataset	Diabetes Obes Metab, 2015, 17: 994-1002
976	Exploratory Dataset	Ann Oncol 2017 Jul 1;28(7):1631-1639
977	Exploratory Dataset	N Engl J Med, 2014, 371: 1877-88
978	Exploratory Dataset	Lancet Oncol 2015 Jun;16(6):619-29

© 2024 Zhang F et al. *JAMA Network Open*.

979	Exploratory Dataset	Oncologist 2016 Sep;21(9):1085-90
980	Exploratory Dataset	Lancet, 2015, 386: 541-51
981	Exploratory Dataset	N Engl J Med 2015; 372:30-39
982	Exploratory Dataset	Am J Chin Med 2013;41(2):263-80
983	Exploratory Dataset	Ann Rheum Dis 2017 Dec;76(12):2001-2008
984	Exploratory Dataset	J Clin Oncol 2016 Sep 1;34(25):3005-13
985	Exploratory Dataset	Diabetes Care 2015 Mar;38(3):376-83
986	Exploratory Dataset	Endocr Pract 2017 Mar;23(3):258-265
987	Exploratory Dataset	Lancet 2017 Mar 25;389(10075):1206-1217
988	Exploratory Dataset	Lancet Oncol, 2016, 17: 200-211
989	Exploratory Dataset	Cardiovasc Drugs Ther 2016 Oct;30(5):473-483
990	Exploratory Dataset	Diabetes Care, 2015, 38: 2018-24
991	Exploratory Dataset	Eur Heart J 2015 Nov 14;36(43):2996-3003
992	Exploratory Dataset	Alcohol Clin Exp Res 2018 Jan;42(1):128-134
993	Exploratory Dataset	J Clin Oncol 2017 Jan 20;35(3):343-351
994	Exploratory Dataset	J Eur Acad Dermatol Venereol 2015 Jun;29(6):1082-90
995	Exploratory Dataset	J Clin Oncol 2018 Aug 10;36(23):2386-2394
996	Exploratory Dataset	N Engl J Med 2015 Jul 9;373(2):123-35
997	Exploratory Dataset	Am Heart J 2015 Jun;169(6):906-915e13
998	Exploratory Dataset	Eur Heart J 2015 May 14;36(19):1186-94
999	Exploratory Dataset	Int J Cardiol 2014 Sep;176(1):55-61
1000	Exploratory Dataset	Lancet Oncol 2015 May;16(5):561-8
1001	Exploratory Dataset	Ann Rheum Dis 2017 May;76(5):831-839
1002	Exploratory Dataset	Lancet 2016 Feb 20;387(10020):770-8

© 2024 Zhang F et al. *JAMA Network Open*.

1003	Exploratory Dataset	Diabetes Obes Metab 2016 Nov;18(11):1134-1137
1004	Exploratory Dataset	Diabetes Care 2015 Nov;38(11):2009-17
1005	Exploratory Dataset	N Engl J Med 2016 May 5;374(18):1754-62
1006	Exploratory Dataset	Ann Rheum Dis 2016 Jun;75(6):1057-64
1007	Exploratory Dataset	Diabetes Obes Metab, 2017, 19: 979-988
1008	Exploratory Dataset	Surg Endosc, 2014, 28: 1641-7
1009	Exploratory Dataset	J Clin Oncol, 2016, 34: 2098-106
1010	Exploratory Dataset	N Engl J Med 2015 Nov 5;373(19):1803-13
1011	Exploratory Dataset	N Engl J Med 2015 Oct 22;373(17):1627-39
1012	Exploratory Dataset	Diabetol Metab Syndr 2017 May 15;9:35
1013	Exploratory Dataset	N Engl J Med 2014 May 22;370(21):1993-2001
1014	Exploratory Dataset	Curr Med Res Opin 2017 Oct;33(10):1861-1868
1015	Exploratory Dataset	Lancet Oncol 2016 Sep;17(9):1248-60
1016	Exploratory Dataset	Ann Rheum Dis 2017 Jan;76(1):79-87
1017	Exploratory Dataset	Cardiovasc Diabetol 2017 Jan 21;16(1):13
1018	Exploratory Dataset	Lancet Oncol 2015; 16: 908-18
1019	Exploratory Dataset	Prostate, 2016, 76: 1519-1527
1020	Exploratory Dataset	Br J Dermatol 2016 Aug;175(2):273-86
1021	Exploratory Dataset	N Engl J Med 2015 Oct;373(14):1318-28
1022	Exploratory Dataset	Lancet Oncol 2016 Dec;17(12):1661-1671
1023	Exploratory Dataset	J Clin Lipidol Nov-Dec 2015;9(6):758-769
1024	Exploratory Dataset	Arthritis Rheumatol 2017 Feb;69(2):277-290
1025	Exploratory Dataset	N Engl J Med 2017 Feb 16;376(7):652-662
1026	Exploratory Dataset	Arthritis Rheumatol 2017 Mar;69(3):506-517

© 2024 Zhang F et al. *JAMA Network Open*.

1027	Exploratory Dataset	Gastroenterology 2017 Jul;153(1):77-86e6
1028	Exploratory Dataset	N Engl J Med, 2017, 377: 352-360
1029	Exploratory Dataset	N Engl J Med, 2016, 375: 1834-1844
1030	Exploratory Dataset	N Engl J Med 2016 Mar 31;374(13):1243-52
1031	Exploratory Dataset	Ann Rheum Dis 2017 Jan;76(1):88-95
1032	Exploratory Dataset	Lancet Oncol 2015 Apr;16(4):375-84
1033	Exploratory Dataset	N Engl J Med 2015; 372:320-330
1034	Exploratory Dataset	N Engl J Med, 2015, 373: 2425-37
1035	Exploratory Dataset	Am J Hematol, 2018, 93: 1402-1410
1036	Exploratory Dataset	J Clin Endocrinol Metab 2015 Aug;100(8):3140-8
1037	Exploratory Dataset	Atherosclerosis 2016 Jan;244:138-46
1038	Exploratory Dataset	N Engl J Med, 2019, 380: 347-357
1039	Exploratory Dataset	Diabetes Care 2017 Feb;40(2):201-209
1040	Exploratory Dataset	N Engl J Med 2016 Nov 17;375(20):1925-1936
1041	Exploratory Dataset	J Dermatol Sci 2016 Jan;81(1):44-52
1042	Exploratory Dataset	Lancet 2015 Sep 19;386(9999):1137-46
1043	Exploratory Dataset	Rheumatology (Oxford) 2017 Nov 1;56(11):1993-2003
1044	Exploratory Dataset	J Crohns Colitis 2016 Jun;10(6):631-40
1045	Exploratory Dataset	Scand J Rheumatol 2018 Jul;47(4):276-281
1046	Exploratory Dataset	Lancet 2017 Jan 7;389(10064):56-66
1047	Exploratory Dataset	Diabetes Obes Metab 2017 Feb;19(2):266-274
1048	Exploratory Dataset	N Engl J Med 2017; 377:317-328
1049	Exploratory Dataset	Clin Microbiol Infect, 2016, 22: 565e1-9
1050	Exploratory Dataset	J Am Acad Dermatol 2017 Jan;76(1):70-80

© 2024 Zhang F et al. *JAMA Network Open*.

1051	Exploratory Dataset	Diabetes Care 2016 Mar;39(3):353-62
1052	Exploratory Dataset	Cardiovasc Diabetol 2017 Feb 23;16(1):27
1053	Exploratory Dataset	Aliment Pharmacol Ther 2016 Jul;44(2):157-69
1054	Exploratory Dataset	J Clin Oncol 2017 Feb 20;35(6):591-597
1055	Exploratory Dataset	J Child Adolesc Psychopharmacol 2017 Feb;27(1):66-74
1056	Exploratory Dataset	Lancet Oncol, 2017, 18: 1261-1273
1057	Exploratory Dataset	N Engl J Med 2015; 373:23-34
1058	Exploratory Dataset	N Engl J Med 2017 Oct 5;377(14):1345-1356
1059	Exploratory Dataset	N Engl J Med 2016 Dec 1;375(22):2154-2164
1060	Exploratory Dataset	Lancet Oncol 2016 Jul;17(7):917-927
1061	Exploratory Dataset	N Engl J Med 2015 Jun 25;372(26):2521-32
1062	Exploratory Dataset	Lancet 2017;390(10105): 1853–1862
1063	Exploratory Dataset	N Engl J Med 2017 Oct 19;377(16):1525-1536
1064	Exploratory Dataset	N Engl J Med, 2018, 379: 2517-2528
1065	Exploratory Dataset	Diabetes Obes Metab 2017 Jun;19(6):892-896
1066	Exploratory Dataset	Ann Rheum Dis 2017 Jun;76(6):1009-1019
1067	Exploratory Dataset	Lancet Oncol 2016 Dec;17(12):1732-1742
1068	Exploratory Dataset	Lancet 2016 Apr 30;387(10030):1837-46
1069	Exploratory Dataset	Lancet 2016 Apr 9;387(10027):1540-1550
1070	Exploratory Dataset	Diabetes Obes Metab, 2016, 18: 1191-1198
1071	Exploratory Dataset	Lancet Oncol 2018 Oct;19(10):1315-1327
1072	Exploratory Dataset	Lancet, 2016, 388: 2128-2141
1073	Exploratory Dataset	JAMA, 2017, 318: 1460-1470
1074	Exploratory Dataset	Lancet Oncol 2016 Nov;17(11):1558-1568

© 2024 Zhang F et al. *JAMA Network Open*.

1075	Exploratory Dataset	N Engl J Med 2015 May 21;372(21):2006-17
1076	Exploratory Dataset	Lancet Oncol 2016 Jul;17(7):883-895
1077	Exploratory Dataset	Lancet, 2016, 388: 2115-2127
1078	Exploratory Dataset	Lancet Diabetes Endocrinol, 2017, 5: 341-354
1079	Exploratory Dataset	Int J Clin Oncol 2015 Oct;20(5):905-12
1080	Exploratory Dataset	Lancet Oncol 2016 Apr;17(4):425-439
1081	Exploratory Dataset	Cardiovasc Diabetol 2015 Jan 30;14:11
1082	Exploratory Dataset	N Engl J Med 2016 Nov 3;375(18):1738-1748
1083	Exploratory Dataset	Diabetes Obes Metab 2017 May;19(5):721-728
1084	Exploratory Dataset	JAMA, 2017, 318: 557-566
1085	Exploratory Dataset	Arthritis Rheumatol 2016 Dec;68(12):2867-2877
1086	Exploratory Dataset	Lancet Infect Dis 2015 Apr;15(4):397-404
1087	Exploratory Dataset	Cancer Med 2018 Apr;7(4):1043-1055
1088	Exploratory Dataset	J Child Adolesc Psychopharmacol 2015 Jun;25(5):402-14
1089	Exploratory Dataset	Diabetes Ther 2018 Feb;9(1):49-66
1090	Exploratory Dataset	Arthritis Res Ther 2018 Mar 15;20(1):47
1091	Exploratory Dataset	Lancet Neurol 2017 Dec;16(12):976-986
1092	Exploratory Dataset	Diabetes Ther 2018 Feb;9(1):193-207
1093	Exploratory Dataset	N Engl J Med, 2018, 378: 2465-2474
1094	Exploratory Dataset	Lancet 2017 Jan 21;389(10066):255-265
1095	Exploratory Dataset	Diabetes Obes Metab, 2016, 18: 803-11
1096	Exploratory Dataset	Arthritis Res Ther 2017 Dec 22;19(1):285
1097	Exploratory Dataset	Neuro Oncol 2018 Apr 9;20(5):674-686
1098	Exploratory Dataset	Lancet 2017 Apr 29;389(10080):1699-1709

© 2024 Zhang F et al. *JAMA Network Open*.

1099	Exploratory Dataset	Diabetes Obes Metab, 2018, 20: 530-540
1100	Exploratory Dataset	Lancet Oncol 2016 Nov;17(11):1497-1508
1101	Exploratory Dataset	N Engl J Med, 2017, 376: 2415-2426
1102	Exploratory Dataset	N Engl J Med, 2018, 378: 1583-1592
1103	Exploratory Dataset	Lancet 2017 Jan 21;389(10066):266-275
1104	Exploratory Dataset	Lancet Diabetes Endocrinol, 2017, 5: 251-260
1105	Exploratory Dataset	Arthritis Rheumatol 2016 Dec;68(12):2857-2866
1106	Exploratory Dataset	N Engl J Med, 2017, 376: 2448-2458
1107	Exploratory Dataset	Ann Allergy Asthma Immunol 2017 Apr;118(4):489-499e1
1108	Exploratory Dataset	J Diabetes 2018 Jul;10(7):589-599
1109	Exploratory Dataset	AIDS 2017 Nov 28;31(18):2503-2514
1110	Exploratory Dataset	N Engl J Med 2016 Nov 10;375(19):1856-1867
1111	Exploratory Dataset	Antivir Ther 2017;22(4):295-305
1112	Exploratory Dataset	Respirology 2014 Nov;19(8):1178-82
1113	Exploratory Dataset	J Clin Oncol 2017 Sep 1;35(25):2875-2884
1114	Exploratory Dataset	Lancet Diabetes Endocrinol, 2017, 5: 355-366
1115	Exploratory Dataset	J Asthma 2018 Jun;55(6):640-650
1116	Exploratory Dataset	Allergy Asthma Proc 2017 Sep 21;38(5):343-353
1117	Exploratory Dataset	N Engl J Med 2016 Nov 10;375(19):1823-1833
1118	Exploratory Dataset	Diabetes Obes Metab, 2017, 19: 1024-1031
1119	Exploratory Dataset	J Diabetes Investig 2016 Jul;7(4):555-64
1120	Exploratory Dataset	Respir Med 2017 Nov;132:31-41
1121	Exploratory Dataset	Rheumatol Ther 2018 Dec;5(2):447-462
1122	Exploratory Dataset	N Engl J Med 2018 Jun 21;378(25):2399-2410

© 2024 Zhang F et al. *JAMA Network Open*.

1123	Exploratory Dataset	Allergy Asthma Proc 2017 Jul 24;38(4):264-276
1124	Exploratory Dataset	Diabetes Obes Metab 2016 Dec;18(12):1207-1216
1125	Exploratory Dataset	Diabetes Obes Metab 2017 Oct;19(10):1397-1407
1126	Exploratory Dataset	N Engl J Med 2015 Dec 31;373(27):2599-607
1127	Exploratory Dataset	Cardiovasc Diabetol 2016 Jun 18;15:89
1128	Exploratory Dataset	Diabetes Care, 2018, 41: 2136-2146
1129	Exploratory Dataset	N Engl J Med 2018 Apr 5;378(14):1277-1290
1130	Exploratory Dataset	N Engl J Med 2017 Mar 16;376(11):1015-1026
1131	Exploratory Dataset	Lancet Oncol 2019 Jan;20(1):43-56
1132	Exploratory Dataset	J Am Acad Dermatol 2017 Oct;77(4):667-674
1133	Exploratory Dataset	Lancet 2017;390(10111):2461–2471
1134	Exploratory Dataset	J Asthma 2018 Sep;55(9):984-993
1135	Exploratory Dataset	Lancet, 2018, 391: 748-757
1136	Exploratory Dataset	Eye (Lond), 2017, 31: 389-394
1137	Exploratory Dataset	Lancet 2018;391(10136):2213-2224
1138	Exploratory Dataset	Lancet Respir Med, 2017, 5: 568-576
1139	Exploratory Dataset	Lancet 2018 Oct 13;392(10155):1330-1339
1140	Exploratory Dataset	Lancet 2017 Jun 10;389(10086):2317-2327
1141	Exploratory Dataset	Diabetes Obes Metab 2017 Jun;19(6):874-882
1142	Exploratory Dataset	J Hepatol 2016 Dec;65(6):1112-1119
1143	Exploratory Dataset	N Engl J Med 2018;378(19):1789–1801
1144	Exploratory Dataset	Lancet, 2018, 392: 123-133
1145	Exploratory Dataset	Lancet Oncol 2018 May;19(5):672-681
1146	Exploratory Dataset	Lancet Oncol, 2018, 19: 1468-1479

© 2024 Zhang F et al. *JAMA Network Open*.

1147	Exploratory Dataset	Ann Rheum Dis 2018 Jun;77(6):890-897
1148	Exploratory Dataset	Lancet 2018 Mar 3;391(10123):839-849 (https://wwwviraledcom/modules/info/files/files_58c03e5ca1c3apdf)
1149	Exploratory Dataset	N Engl J Med, 2019, 381: 121-131
1150	Exploratory Dataset	N Engl J Med 2018 May 31;378(22):2093-2104
1151	Exploratory Dataset	Lancet Oncol 2018 Mar;19(3):416-426
1152	Exploratory Dataset	N Engl J Med 2018;378(22):2078–2092
1153	Exploratory Dataset	Lancet 2018 Jun 23;391(10139):2503-2512
1154	Exploratory Dataset	J Clin Oncol, 2019, 37: 2974-2986
1155	Exploratory Dataset	Lancet 2018 Dec 8;392(10163):2441-2451
1156	Exploratory Dataset	Arthritis Rheumatol 2019 Apr;71(4):599-611
1157	Exploratory Dataset	Lancet 2018 Jun 23;391(10139):2513-2524
1158	Exploratory Dataset	J Periodontol, 2017, 88: 1244-1252
1159	Exploratory Dataset	J Dent, 2018, 72: 76-79
1160	Exploratory Dataset	J Clin Oncol 2016 Aug 10;34(23):2728-35
1161	Exploratory Dataset	Lancet Infect Dis, 2016, 16: 905-14
1162	Exploratory Dataset	Int J Tuberc Lung Dis 2012 Feb;16(2):268-72
1163	Exploratory Dataset	BMC Cancer 2015 May 11;15:392
1164	Exploratory Dataset	Curr Med Res Opin 2014 Jul;30(7):1231-44
1165	Exploratory Dataset	Thorax 2012 Aug;67(8):689-93
1166	Exploratory Dataset	J Clin Oncol, 2010, 28: 3191-8
1167	Exploratory Dataset	J Clin Endocrinol Metab 2009 Feb;94(2):538-44
1168	Exploratory Dataset	J Clin Endocrinol Metab 2012 Jan;97(1):286-92
1169	Exploratory Dataset	Lancet, 2012, 380: 660-7

© 2024 Zhang F et al. *JAMA Network Open*.

1170	Exploratory Dataset	Ann Rheum Dis 2014 Jun;73(6):1044-51
1171	Exploratory Dataset	J Child Psychol Psychiatry 2015 Apr;56(4):444-52
1172	Exploratory Dataset	JAMA, 2013, 309: 1260-7
1173	Exploratory Dataset	Lancet 2017;390(10095):659-68
1174	Exploratory Dataset	J Negat Results Biomed, 2015, 14: 15
1175	Exploratory Dataset	Inflamm Bowel Dis 2015 Oct;21(10):2247-53
1176	Exploratory Dataset	Gastroenterology 2013 Oct;145(4):766-74e1
1177	Exploratory Dataset	J Pain Symptom Manage, 2015, 49: 161-72
1178	Exploratory Dataset	Lancet Oncol 2014 Jul;15(8):862-73
1179	Exploratory Dataset	Osteoarthritis Cartilage, 2013, 21: 22-7
1180	Exploratory Dataset	Cancer Prev Res (Phila) 2011 Aug;4(8):1181-9
1181	Exploratory Dataset	Arthritis Res Ther 2012 Oct 24;14(5):R230
1182	Exploratory Dataset	Lancet Oncol 2013 May;14(6):490-9
1183	Exploratory Dataset	Lancet Oncol 2017 Apr;18(4):486-499
1184	Exploratory Dataset	Laryngoscope, 2015, 125(5):1048-1055
1185	Exploratory Dataset	Lancet Infect Dis, 2017, 17: 322-329
1186	Exploratory Dataset	J Clin Oncol 2012 May 1;30(13):1484-91
1187	Exploratory Dataset	Ther Adv Respir Dis Jan-Dec 2018;12:1753466618777924
1188	Exploratory Dataset	Diabetes Obes Metab 2010 Sep;12(9):780-9
1189	Exploratory Dataset	Allergy, 2011, 66: 1457-68
1190	Exploratory Dataset	Diabetes Obes Metab 2013 Dec;15(12):1111-9
1191	Exploratory Dataset	Diabetes Obes Metab 2015 Jul;17(7):699-702
1192	Exploratory Dataset	Int J Hematol Oncol Stem Cell Res, 2018, 12: 77-83
1193	Exploratory Dataset	Br J Cancer 2016 Nov 8;115(10):1193-1200

© 2024 Zhang F et al. *JAMA Network Open*.

1194	Exploratory Dataset	J Child Psychol Psychiatry 2013 May;54(5):527-35
1195	Exploratory Dataset	Lancet Oncol, 2012, 13: 1161-70
1196	Exploratory Dataset	Rheumatology (Oxford) 2012;51:1368-77
1197	Exploratory Dataset	Health Technol Assess 2013 Feb;17(4):1-218
1198	Exploratory Dataset	Breast Cancer Res Treat 2010 Jul;122(2):429-37
1199	Exploratory Dataset	Lancet 2013 Mar 9;381(9869):805-16
1200	Exploratory Dataset	Lancet Oncol 2015 Nov;16(15):1515-1524
1201	Exploratory Dataset	BMC Fam Pract, 2002, 3: 10
1202	Exploratory Dataset	Lancet 2011 Jun 18;377(9783):2103-14
1203	Exploratory Dataset	Lancet Oncol 2014 Jul;15(8):894-904
1204	Exploratory Dataset	Ann Rheum Dis 2013 Jun;72(6):844-50
1205	Exploratory Dataset	Lancet Oncol 2013 Jun;14(7):627-37
1206	Exploratory Dataset	Ann Intern Med, 2008, 148: 268-77
1207	Exploratory Dataset	Chest, 2016, 149: 1052-60
1208	Exploratory Dataset	N Engl J Med 2006 Feb 23;354(8):809-20
1209	Exploratory Dataset	Lancet Oncol, 2014, 15: 620-30
1210	Exploratory Dataset	Br J Cancer 2014 Apr 29;110(9):2178-86
1211	Exploratory Dataset	PLoS Med, 2009, 6: e1000191
1212	Exploratory Dataset	Lancet 2010 Jan 30;375(9712):377-84
1213	Exploratory Dataset	N Engl J Med, 2011, 365: 2484-96
1214	Exploratory Dataset	Lancet Oncol 2013 Jul;14(8):749-59
1215	Exploratory Dataset	Eur Respir J 2008 Sep;32(3):619-28
1216	Exploratory Dataset	Cardiovasc Diabetol 2014 Mar 28;13:65
1217	Exploratory Dataset	Lung Cancer 2012 Jun;76(3):362-7

© 2024 Zhang F et al. *JAMA Network Open*.

1218	Exploratory Dataset	Lancet Oncol 2013 Dec;14(13):1278-86
1219	Exploratory Dataset	Gastric Cancer 2015 Oct;18(4):824-32
1220	Exploratory Dataset	Adv Ther 2015 Apr;32(4):319-40
1221	Exploratory Dataset	Schizophr Res 2015 Feb;161(2-3):421-8
1222	Exploratory Dataset	Aliment Pharmacol Ther 2013 Aug;38(3):264-73
1223	Exploratory Dataset	Curr Med Res Opin 2014 Jul;30(7):1245-55
1224	Exploratory Dataset	Gastroenterology 2015 Dec;149(7):1775-1783e2
1225	Exploratory Dataset	Curr Med Res Opin 2018 Jun;34(6):981-994
1226	Exploratory Dataset	Radiother Oncol 2014 Jan;110(1):126-31
1227	Exploratory Dataset	Breast Cancer Res Treat 2010 Jun;121(2):379-87
1228	Exploratory Dataset	Br J Cancer 2012 Jun 5;106(12):1934-9
1229	Exploratory Dataset	Lancet Oncol 2010 Feb;11(2):121-8
1230	Exploratory Dataset	Ann Oncol 2015 Jul;26(7):1401-8
1231	Exploratory Dataset	Eur J Haematol, 2016, 96: 602-9
1232	Exploratory Dataset	J Infect, 2010, 61: 410-8
1233	Exploratory Dataset	Lung Cancer 2015 Aug;89(2):146-53
1234	Exploratory Dataset	Cancer Sci 2016 Dec;107(12):1843-1850
1235	Exploratory Dataset	Brain 2015 Nov;138(Pt 11):3400-12
1236	Exploratory Dataset	Front Psychiatry 2016 Jan 21;7:2
1237	Exploratory Dataset	Dig Dis Sci 2018 Jul;63(7):1910-1919
1238	Exploratory Dataset	J Clin Oncol 2010 Feb 10;28(5):753-60
1239	Exploratory Dataset	J Diabetes Investig 2014 May 4; 5(3): 320–326
1240	Exploratory Dataset	Diabetes Care 2017 Oct;40(10):1364-1372
1241	Exploratory Dataset	Ann Oncol 2008 Jul;19(7):1288-1292

© 2024 Zhang F et al. *JAMA Network Open*.

1242	Exploratory Dataset	Diabetes Obes Metab 2009 Jun;11(6):589-95
1243	Exploratory Dataset	Inflamm Bowel Dis 2017 Feb;23(2):261-271
1244	Exploratory Dataset	Alcohol Clin Exp Res 2007 Apr;31(4):625-34
1245	Exploratory Dataset	N Engl J Med 2005 Apr 21;352(16):1637-45
1246	Exploratory Dataset	N Engl J Med, 2000, 342: 1085-92
1247	Exploratory Dataset	J Clin Oncol 2008 Oct 20;26(30):4883-90
1248	Exploratory Dataset	J Clin Oncol 2006 Jul 1;24(19):3121-7
1249	Exploratory Dataset	J Clin Oncol 2003 Aug 15;21(16):3025-34
1250	Exploratory Dataset	J Am Acad Child Adolesc Psychiatry 2006 Nov;45(11):1294-1303
1251	Exploratory Dataset	J Clin Oncol 2007 Apr 20;25(12):1539-44
1252	Exploratory Dataset	N Engl J Med, 2005, 353: 123-32
1253	Exploratory Dataset	Diabetes Care 2004 Nov;27(11):2628-35
1254	Exploratory Dataset	Gastroenterology 2006 Feb;130(2):323-33; quiz 591
1255	Exploratory Dataset	J Clin Oncol 2007 Sep 20;25(27):4278-84
1256	Exploratory Dataset	J Clin Oncol 2009 Jul 10;27(20):3312-8
1257	Exploratory Dataset	Oncologist 2010;15(2):122-9
1258	Exploratory Dataset	J Clin Oncol 2008 Dec 1;26(34):5544-52
1259	Exploratory Dataset	Gastroenterology 2007 Jan;132(1):52-65
1260	Exploratory Dataset	JAMA 2010 Nov 17;304(19):2154-60
1261	Exploratory Dataset	Int J Clin Pract 2005 Dec;59(12):1377-86
1262	Exploratory Dataset	Pediatr Infect Dis J 2007 Mar;26(3):201-9
1263	Exploratory Dataset	Int J Cardiol 2005 Jul 10;102(2):327-32
1264	Exploratory Dataset	Int J Clin Pract 2005 Jun;59(6):619-27
1265	Exploratory Dataset	J Clin Oncol 2009 Jul 10;27(20):3385-90

© 2024 Zhang F et al. *JAMA Network Open*.

1266	Exploratory Dataset	Diabet Med 2010 Mar;27(3):318-26
1267	Exploratory Dataset	Drug Alcohol Depend 2010 Oct 1; 111(3): 200–206
1268	Exploratory Dataset	Diabetes Obes Metab 2007 Sep;9(5):733-45
1269	Exploratory Dataset	Lancet, 2005, 366: 1367-74
1270	Exploratory Dataset	J Am Acad Dermatol . 2007 Jan;56(1):31.e1-15
1271	Exploratory Dataset	J Clin Oncol 2012 May 1;30(13):1534-40
1272	Exploratory Dataset	J Thorac Oncol 2010 Dec;5(12):1977-85
1273	Exploratory Dataset	J Clin Oncol 2007 May 1;25(13):1658-64
1274	Exploratory Dataset	J Clin Oncol 2009 Feb 10;27(5):672-80
1275	Exploratory Dataset	J Clin Oncol 2009 Sep 20;27(27):4487-91
1276	Exploratory Dataset	J Clin Oncol 2009 Feb 10;27(5):663-71
1277	Exploratory Dataset	Rheumatology (Oxford) 2007 Jul;46(7):1122-5
1278	Exploratory Dataset	Lung Cancer 2008 Dec;62(3):334-43
1279	Exploratory Dataset	Int J Gynecol Cancer Sep-Oct 2008;18(5):1013-9
1280	Exploratory Dataset	Ann Oncol 2008 Apr;19(4):734-8
1281	Exploratory Dataset	Neurology 2008 Jul 22;71(4):265-71
1282	Exploratory Dataset	J Am Acad Dermatol, 2004, 51: 534-42
1283	Exploratory Dataset	J Clin Oncol 2010 Jan 1;28(1):49-55
1284	Exploratory Dataset	Eur Neuropsychopharmacol 2005 Jan;15(1):111-7
1285	Exploratory Dataset	Arch Pediatr Adolesc Med 2006 Jan;160(1):82-90
1286	Exploratory Dataset	Clin Colorectal Cancer 2012 Jun;11(2):101-11
1287	Exploratory Dataset	Mayo Clin Proc, 2005, 80: 470-9
1288	Exploratory Dataset	Pediatrics 2001 Oct;108(4):883-92
1289	Exploratory Dataset	J Clin Psychopharmacol 2012 Oct;32(5):630-6

© 2024 Zhang F et al. *JAMA Network Open*.

1290	Exploratory Dataset	Am J Cardiol 2008 Dec 1;102(11):1489-94
1291	Exploratory Dataset	J Med Econ, 2013, 16: 1061-70
1292	Exploratory Dataset	J Clin Oncol 2010 Apr 10;28(11):1835-42
1293	Exploratory Dataset	J Child Adolesc Psychopharmacol 2009 Aug;19(4):351-61
1294	Exploratory Dataset	Arthritis Rheum 2004 Jul;50(7):2264-72
1295	Exploratory Dataset	J Thorac Oncol, 2013, 8: 79-88
1296	Exploratory Dataset	Gastroenterology 2012 May;142(5):1102-1111.e2
1297	Exploratory Dataset	Diabetes Obes Metab 2009 Dec;11(12):1153-62
1298	Exploratory Dataset	Ann Oncol 2014 Jul;25(7):1346-1355
1299	Exploratory Dataset	Clinical Therapeutics 2013;35(7):431-7
1300	Exploratory Dataset	Int Clin Psychopharmacol 2015
1301	Exploratory Dataset	Aliment Pharmacol Ther 2007 Aug 1;26(3):421-30
1302	Exploratory Dataset	Ann Oncol, 2013, 24: 702-9
1303	Exploratory Dataset	Arthritis Rheum, 2001, 44: 2539-47
1304	Exploratory Dataset	Prostate Cancer Prostatic Dis 2012 Mar;15(1):87-92
1305	Exploratory Dataset	Clin Gastroenterol Hepatol 2007 Jan;5(1):95-102
1306	Exploratory Dataset	J Bone Miner Res 2011 Jun;26(6):1303-12
1307	Exploratory Dataset	Gastroenterology 2007 Jan;132(1):66-75; quiz 432-3
1308	Exploratory Dataset	Am J Cardiol 2004 Jun 15;93(12):1481-6
1309	Exploratory Dataset	Clin Ther 2007 Mar;29(3):450-63
1310	Exploratory Dataset	Heart Rhythm, 2012, 9: 172-8
1311	Exploratory Dataset	Nicotine Tob Res 2011 Oct;13(10):955-64
1312	Exploratory Dataset	J Bone Miner Res 2009 Apr;24(4):719-25
1313	Exploratory Dataset	J Rheumatol 2007 May;34(5):1040-50

© 2024 Zhang F et al. *JAMA Network Open*.

1314	Exploratory Dataset	J Cardiovasc Med (Hagerstown) 2008 Jul;9(7):688-93
1315	Exploratory Dataset	J Thorac Oncol, 2014, 9: 214-21
1316	Exploratory Dataset	J Allergy Clin Immunol 2005 Dec;116(6):1289-95
1317	Exploratory Dataset	J Thorac Oncol 2014 May;9(5):733-7
1318	Exploratory Dataset	Ann Rheum Dis 2013 Sep 1;72(9):1488-95
1319	Exploratory Dataset	J Am Acad Dermatol, 2012, 67: 86-92
1320	Exploratory Dataset	Lung Cancer, 2016, 102: 65-73
1321	Exploratory Dataset	Eur Neuropsychopharmacol 2013 Nov;23(11):1432-42
1322	Exploratory Dataset	Nicotine Tob Res 2011 Sep;13(9):820-6
1323	Exploratory Dataset	Ann Oncol 2013 Mar;24(3):718-25
1324	Exploratory Dataset	J Gastroenterol 2014 Feb;49(2):283-94
1325	Exploratory Dataset	Diabetes Obes Metab 2012 Oct;14(10):910-7
1326	Exploratory Dataset	J Drugs Dermatol 2013;12:166–74
1327	Exploratory Dataset	Nicotine Tob Res 2014 Jan;16(1):50-7
1328	Exploratory Dataset	Am J Gastroenterol 2016 Nov;111(11):1599-1607
1329	Exploratory Dataset	Chin Med J (Engl), 2012, 125: 1845-51
1330	Exploratory Dataset	J Dermatolog Treat, 2014, 25: 57-60
1331	Exploratory Dataset	Curr Med Res Opin, 2010, 26: 1277-84
1332	Exploratory Dataset	Dermatol Surg, 2014, 40: 1181-90
1333	Exploratory Dataset	Diabetes Obes Metab 2014 May;16(5):403-9
1334	Exploratory Dataset	Diabetes Obes Metab 2014 Nov;16(11):1102-10
1335	Exploratory Dataset	Arthritis Rheumatol 2016 Jan;68(1):46-55
1336	Exploratory Dataset	J Neurooncol 2017 Jan;131(1):105-115
1337	Exploratory Dataset	J Clin Hypertens (Greenwich) 2016 Jan;18(1):43-52

© 2024 Zhang F et al. *JAMA Network Open*.

1338	Exploratory Dataset	Diabetes Obes Metab 2016 Aug;18(8):812-9
1339	Exploratory Dataset	N Engl J Med, 2005, 352: 1425-35
1340	Exploratory Dataset	Oral Oncol 2014 May;50(5):498-505
1341	Validation Dataset	N Engl J Med. 2007 Aug 30;357(9):874-84
1342	Validation Dataset	J Assoc Physicians India. 2008 Jun:56:418-24
1343	Validation Dataset	J Am Coll Surg. 2009 Jan;208(1):1-13
1344	Validation Dataset	Obstet Gynecol. 2008 Mar;111(3):639-47
1345	Validation Dataset	Rev Esp Cardiol. 2009 Apr;62(4):400-8
1346	Validation Dataset	Vaccine. 2009 Jan 29;27(5):765-72
1347	Validation Dataset	Lancet. 2009 Apr 4;373(9670):1183-9
1348	Validation Dataset	Kardiol Pol. 2009 Feb;67(2):123-7; discussion 128-9
1349	Validation Dataset	Circulation. 2010 Jan 5;121(1):143-50
1350	Validation Dataset	Lancet Oncol. 2011 Jan;12(1):30-7
1351	Validation Dataset	N Engl J Med. 2010 Dec 9;363(24):2310-9
1352	Validation Dataset	J Bone Miner Res. 2010 Nov;25(11):2368-73
1353	Validation Dataset	Ann Intern Med. 2010 Jun 15;152(12):761-9
1354	Validation Dataset	Ann Surg. 2011 Mar;253(3):495-501
1355	Validation Dataset	Am J Med. 2011 May;124(5):434-43
1356	Validation Dataset	J Clin Psychiatry. 2011 Feb;72(2):168-74
1357	Validation Dataset	Eur Neuropsychopharmacol. 2011 Mar;21(3):221-9
1358	Validation Dataset	Clin Ther. 2012 Jan;34(1):77-90
1359	Validation Dataset	Psychosom Med. 2012 Jul-Aug;74(6):628-34
1360	Validation Dataset	ANZ J Surg. 2012 Sep;82(9):630-2
1361	Validation Dataset	Am J Obstet Gynecol. 2012 May;206(5):431.e1-5

© 2024 Zhang F et al. *JAMA Network Open*.

1362	Validation Dataset	Blood. 2012 Sep 27;120(13):2581-8
1363	Validation Dataset	Diabetologia. 2012 Jan;55(1):105-13
1364	Validation Dataset	J Clin Psychiatry. 2012 Jul;73(7):984-91
1365	Validation Dataset	Lancet Oncol. 2013 Dec;14(13):1317-25
1366	Validation Dataset	N Engl J Med. 2013 Sep 12;369(11):1023-34
1367	Validation Dataset	Antiinflamm Antiallergy Agents Med Chem. 2013;12(3):223-8
1368	Validation Dataset	J Aerosol Med Pulm Drug Deliv. 2014 Apr;27(2):133-7
1369	Validation Dataset	Int J Antimicrob Agents. 2013 Jan;41(1):57-64
1370	Validation Dataset	J Psychiatr Res. 2013 Jan;47(1):113-21
1371	Validation Dataset	Ann Oncol. 2013 Jun;24(6):1505-12
1372	Validation Dataset	J Cardiovasc Electrophysiol. 2013 Apr;24(4):442-8
1373	Validation Dataset	Surg Endosc. 2015 Mar;29(3):692-9
1374	Validation Dataset	Pain. 2014 Dec;155(12):2510-2516
1375	Validation Dataset	J Am Coll Cardiol. 2014 Jun 17;63(23):2560-2568
1376	Validation Dataset	Sleep Med. 2014 Oct;15(10):1276-8
1377	Validation Dataset	Vaccine. 2015 Feb 11;33(7):933-41
1378	Validation Dataset	Diabetes Care. 2014 Feb;37(2):e28-9
1379	Validation Dataset	Dement Geriatr Cogn Disord. 2014;37(3-4):232-45
1380	Validation Dataset	Ocul Surf. 2014 Apr;12(2):146-54
1381	Validation Dataset	N Engl J Med. 2014 Aug 21;371(8):711-22
1382	Validation Dataset	Aliment Pharmacol Ther. 2015 Jun;41(11):1132-40
1383	Validation Dataset	Am J Prev Med. 2015 Jan;48(1):1-12
1384	Validation Dataset	Appetite. 2015 Jan:84:154-60
1385	Validation Dataset	J Card Fail. 2016 Jul;22(7):529-36

© 2024 Zhang F et al. *JAMA Network Open*.

1386	Validation Dataset	Hypertension. 2015 Feb;65(2):313-9
1387	Validation Dataset	Eur J Cancer. 2015 Jan;51(1):45-54
1388	Validation Dataset	Am Heart J. 2016 Feb:172:88-95
1389	Validation Dataset	Ann Oncol. 2015 Feb;26(2):313-20
1390	Validation Dataset	Surg Endosc. 2016 Jan;30(1):204-14
1391	Validation Dataset	Cancer. 2016 Jan 15;122(2):287-95
1392	Validation Dataset	Contemp Clin Trials. 2016 Sep:50:45-53
1393	Validation Dataset	J Clin Oncol. 2016 Jun 10;34(17):2046-53
1394	Validation Dataset	Am J Respir Crit Care Med. 2016 Aug 15;194(4):486-92
1395	Validation Dataset	Nutr Metab Cardiovasc Dis. 2016 Apr;26(4):302-9
1396	Validation Dataset	Chin Med J (Engl). 2016 Nov 20;129(22):2647-2651
1397	Validation Dataset	Ann Behav Med. 2016 Oct;50(5):751-761
1398	Validation Dataset	J Rehabil Res Dev. 2016;53(1):107-16
1399	Validation Dataset	Eur J Cancer. 2016 Jan:52:173-80
1400	Validation Dataset	Am J Sports Med. 2016 Dec;44(12):3119-3125
1401	Validation Dataset	Gastroenterology. 2018 Feb;154(3):529-539.e2
1402	Validation Dataset	Eur J Prev Cardiol. 2017 Sep;24(14):1544-1554
1403	Validation Dataset	Circ Cardiovasc Interv. 2017 Apr;10(4):e004460
1404	Validation Dataset	Zhonghua Xin Xue Guan Bing Za Zhi. 2017 Mar 24;45(3):190-197
1405	Validation Dataset	J Clin Oncol. 2018 Jun 10;36(17):1658-1667
1406	Validation Dataset	Arch Phys Med Rehabil. 2017 Aug;98(8):1499-1507.e2
1407	Validation Dataset	Ann Rheum Dis. 2018 Feb;77(2):212-220
1408	Validation Dataset	Aliment Pharmacol Ther. 2017 Feb;45(3):391-402
1409	Validation Dataset	Ann Allergy Asthma Immunol. 2017 Aug;119(2):189-190

© 2024 Zhang F et al. *JAMA Network Open*.

1410	Validation Dataset	J Child Adolesc Psychopharmacol. 2018 Jun;28(5):322-330
1411	Validation Dataset	Interact Cardiovasc Thorac Surg. 2018 May 1;26(5):790-797
1412	Validation Dataset	Lancet Gastroenterol Hepatol. 2018 Dec;3(12):845-855
1413	Validation Dataset	N Engl J Med. 2018 Apr 5;378(14):1277-1290
1414	Validation Dataset	Blood Adv. 2018 Nov 13;2(21):2837-2847
1415	Validation Dataset	Eur J Nutr. 2018 Feb;57(1):243-250
1416	Validation Dataset	Ann Rheum Dis. 2018 Dec;77(12):1710-1719
1417	Validation Dataset	Haematologica. 2018 Dec;103(12):2079-2087
1418	Validation Dataset	Diabet Med. 2018 Apr;35(4):483-490
1419	Validation Dataset	Int J Obes (Lond). 2019 Jul;43(7):1485-1490
1420	Validation Dataset	Probiotics Antimicrob Proteins. 2019 Dec;11(4):1195-1201
1421	Validation Dataset	J Trauma Acute Care Surg. 2019 Feb;86(2):181-188
1422	Validation Dataset	J Am Pharm Assoc (2003). 2020 Mar-Apr;60(2):344-351.e2
1423	Validation Dataset	Headache. 2019 Jul;59(7):1052-1062
1424	Validation Dataset	Thorax. 2019 Feb;74(2):197-199
1425	Validation Dataset	Blood. 2019 Mar 7;133(10):1011-1019
1426	Validation Dataset	Thromb Haemost. 2019 Jul;119(7):1171-1181
1427	Validation Dataset	Lancet Oncol. 2020 Jan;21(1):162-174
1428	Validation Dataset	Br J Ophthalmol. 2020 Sep;104(9):1223-1227
1429	Validation Dataset	J Consult Clin Psychol. 2019 Apr;87(4):370-379
1430	Validation Dataset	Am J Cardiol. 2020 Feb 15;125(4):485-490
1431	Validation Dataset	JACC Heart Fail. 2020 May;8(5):359-368
1432	Validation Dataset	Eur J Nutr. 2020 Apr;59(3):1163-1170
1433	Validation Dataset	Med Gas Res. 2020 Jan-Mar;10(1):1-7

© 2024 Zhang F et al. *JAMA Network Open*.

1434	Validation Dataset	JAMA Otolaryngol Head Neck Surg. 2020 Nov 1;146(11):1006-1014
1435	Validation Dataset	Cephalalgia. 2020 Nov;40(13):1523-1531
1436	Validation Dataset	Lancet. 2020 Apr 11;395(10231):1217-1224
1437	Validation Dataset	Ann Neurol. 2020 Aug;88(2):264-273
1438	Validation Dataset	Lancet. 2020 Dec 5;396(10265):1807-1816

© 2024 Zhang F et al. *JAMA Network Open*.

eTable 4. List of excluded studies in validation dataset (with reasons)

Exclusion list		Reasons for exclusion
1	Early experiences and predictors of recruitment success for the National Children's Study. Pediatrics. 2011 Feb;127(2):261-8. doi: 10.1542/peds.2010-2334. Epub 2011 Jan 24.	This study, identified by PubMed as RCT, should be a cohort study.
2	The role of conventional bronchoscopy in the workup of suspicious CT scan screen-detected pulmonary nodules. Chest. 2012 Aug;142(2):377-384. doi: 10.1378/chest.11-2030.	This study, identified by PubMed as RCT, should be a cross-sectional study.

© 2024 Zhang F et al. *JAMA Network Open*.
