# Supplementary material for: Discordant Information on Blinding in Trial Registries and Published Research: A Systematic Review
Source: JAMA Netw Open. 2024 Dec 26;7(12):e2452274. doi: 10.1001/jamanetworkopen.2024.52274 (PMC11672156; doi:10.1001/jamanetworkopen.2024.52274)
Supplement: Supplement 2. — Data Sharing Statement [file jamanetwopen-e2452274-s002.pdf]

Data Sharing Statement

Zhang. Discordant Information on Blinding in Trial Registries and Research Papers. *JAMA Netw Open*. Published December 26, 2024. doi:10.1001/jamanetworkopen.2024.52274

Data
Data available: Yes
Data types: Data (not involving human participants)
How to access data: The datasets used and analysed during the current study are available from the corresponding author on reasonable request.Email: wu_yunhong@163.com
When available: With publication

Supporting Documents
Document types: None

Additional Information
Who can access the data: anyone requesting the data
Types of analyses: on reasonable request
Mechanisms of data availability: with a signed data access agreement
